# Occlusal Splint Therapy in the Management of Temporomandibular Disorders—Evidence from Systematic Reviews

**DOI:** 10.1111/joor.70208

**Published:** 2026-04-30

**Authors:** Daniela Del Sorbo, Tessa Bijelic, Birgitta Häggman‐Henrikson, Thomas List, Ambrosina Michelotti, Rosaria Bucci

**Affiliations:** ^1^ Department of Neuroscience, Reproductive Science and Oral Science University of Naples Federico II Naples Italy; ^2^ Department of Orofacial Pain and Jaw Function, Faculty of Odontology Malmö University Malmö Sweden

**Keywords:** occlusal splint, systematic review, temporomandibular disorders, TMD pain, umbrella review

## Abstract

**Background and Aim:**

Occlusal splints are commonly used to manage temporomandibular disorders (TMDs), yet their efficacy remains controversial. This study aimed to summarise and assess the methodological quality of available systematic reviews (SRs) regarding occlusal splint therapy in TMD patients.

**Materials and Methods:**

Following a PROSPERO‐registered protocol (CRD42021276856), a comprehensive search was conducted across PubMed, Scopus, Lilacs, and Cochrane. SRs focused on TMD management with occlusal splints were included. Two independent operators performed the review process, and the risk of bias was assessed using AMSTAR‐2. Primary outcomes were pain intensity and maximum mouth opening (MMO).

**Results:**

After screening of 1740 abstracts followed by full text assessment, 21 SRs were included with diverse methodological quality ranging from critically low (5 SRs) to high (4 SRs). The majority of the included SRs (12) compared occlusal splint with other conservative treatments, with four of these reporting effective reduction in pain intensity, while three SRs did not. Furthermore, five SRs concluded that there was insufficient evidence for or against occlusal splint therapy over other active interventions for TMD treatment. Only one SR compared the effect of occlusal splint versus no treatment, supporting no differences in pain reduction in the jaw joint area. One SR compared the effects of different occlusal splint designs. Three SRs assessed the effects of occlusal splint in specific treatment settings or populations (in adolescents, compared to arthrocentesis, compared to jaw exercises).

**Conclusion:**

The majority of findings from the existing SRs report small beneficial or neutral effects following OS therapy in TMD patients.

## Graphical abstract

This umbrella review of 21 systematic reviews evaluates occlusal splint therapy for TMD. Findings indicate mostly small beneficial or neutral effects on pain and mouth opening. Consequently, clinicians should prioritise cost‐effectiveness and patient preferences when considering splints, given the high variability in methodological quality across existing evidence.

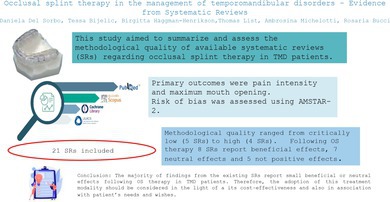

## 1 Introduction

Temporomandibular disorders (TMDs) are one of the most common orofacial pain conditions (prevalence of 5% to 12% [1]) that affect masticatory muscles, temporomandibular joints (TMJs), and the associated structures [1, 2]. The primary symptoms of TMDs include facial and preauricular pain, limited jaw movement, and TMJ noises [3].

Several risk factors have been identified in the development of TMDs, including comorbid painful conditions, psychosocial disturbances, sleep disorders, microtrauma and macrotrauma, and gender‐related predispositions [4]. TMD shares many characteristics with other musculoskeletal pain conditions and is therefore best approached from a biopsychosocial perspective. In line with this model, a range of treatment modalities has been proposed [5], with conservative and non‐invasive options often recommended as first‐line interventions due to their safety and ease of implementation [6]. These interventions include patient education and counselling, pharmacotherapy, manual therapy, therapeutic exercises, and occlusal splint (OS) therapy [7].

OS is one of the most common treatments used for TMD management [8]. There are various designs of OS and among all of them, the stabilisation splint, also called Michigan splint, is the most frequently used [9]. OS are widely used to manage bruxism and to alleviate pain associated with muscular and joint pain attributed to TMDs [10]. However, the precise mechanism of action of OS is unknown. It has been claimed that OS determines a variation in the condylar position, eliminates the occlusal interferences, changes the neuromuscular activity of the masticatory muscles, and increases the vertical dimension of occlusion [11]. These factors may contribute to the reduction of muscle tension and, together with the placebo effect, seem to produce an improvement in symptoms [9]. However, the evidence regarding the efficacy of OS in reducing pain and dysfunction in TMD patients is ambivalent [12].

In recent years, there has been a noticeable increase in the publication of Systematic Reviews (SRs) in every field of dentistry, leading to the subsequent publication of overviews aimed at summarising the findings from multiple SRs [13, 14]. Umbrella Reviews represent a logical and appropriate progression in the field, allowing for the comparison of findings from separate reviews and providing clinical decision‐makers with valuable evidence. The primary goal of such overviews is to identify and evaluate all published systematic reviews, describing their quality, summarising and comparing their results, and discussing the strength of these conclusions. This process ensures that a summary of evidence is accessible from one single paper to clinical decision‐makers. Furthermore, overviews offer a quality assessment of existing SRs on a given topic, describing the current body of evidence and highlighting any research gaps in the existing literature [15].

This umbrella review is part of a larger project aiming at summarising, analysing, and critically appraising the existing SRs on all types of treatment modalities for TMD management [16]. In particular, the current overview focused on the effects of OS in the management of TMDs, assessing also the quality of the available evidence on this topic.

## 2 Method

The present study protocol, registered in the PROSPERO International Prospective Register of Systematic Reviews (CRD42021276856), was developed before the literature search, and the review was performed in accordance with the PRISMA (Preferred Reporting Items for Systematic Reviews and Meta‐analyses) statement [17] and PRIOR guidelines [18].

### 2.1 Eligibility Criteria

The purpose of this umbrella review was to evaluate the evidence for pain relief and functional improvement in TMD following any kind of treatment for TMD [16]. For the purpose of this manuscript, SRs concerning OS therapy were extrapolated. According to the PICOS model, inclusion and exclusion criteria were defined as follows: [19].

*Population* (*P*)*:* Adults and adolescent patients with a diagnosis of TMD pain and/or dysfunction according to the Research Diagnostic Criteria for TMD (RDC/TMD), the Diagnostic Criteria for TMD (DC/TMD), and other diagnostic systems.

*Intervention* (*I*): OS therapy, with any device design.

*Comparison* (*C*): No treatment, waiting list, placebo, or other therapies in single or multiple modalities.

*Outcomes* (*O*): pain intensity (measured with NRS, VAS, or other) and mouth opening. Secondary outcomes were: global improvement, quality of life, and adverse effects.

Study design (S): SRs with or without a meta‐analysis.

The exclusion criteria were: reviews where only one database was used for the electronic literature search and the study selection was not performed systematically, wrong study population (not TMDs), wrong intervention (not on OS treatment), updated or withdrawn reviews, SRs on TMD diagnosis included in the expanded taxonomy [20]. SRs were excluded if the full text was unavailable. The inclusion was limited to publications written in English, German, French, Italian, Spanish, Portuguese, and Swedish.

### 2.2 Information Sources and Search Strategy

The literature search was conducted by one senior librarian (Martina Vall), using two blocks of keywords, MeSH terms and free text terms: the first block included terms that describe TMDs, the second block included terms that describe SR (for detailed search strategies for each database, please see search strings, Appendix S1). The electronic search was conducted on four databases: PubMed (NLM), Scopus (Elsevier), Lilacs (VHL), and Cochrane (Wiley), and complemented with a hand search. Publications in languages other than English, Italian, Spanish, Portuguese, German, Swedish, and French were included in the search and title/abstract screening but excluded during the full‐text assessment. The first search was done in September 2021 and updated on 1 December 2023. Authors were contacted to obtain full texts that were not available on the electronic databases or through other searches. Whenever the full text was not obtained, the study was excluded.

### 2.3 Selection Process

Search results were imported into Covidence (www.covidence.org), which was used for the screening process. Two authors (DDS and TB) independently screened the studies' titles and abstracts. Any disagreement between the two authors was solved by discussion or by consulting a senior reviewer (RB or BHH). The same authors performed the full‐text assessment independently to determine if articles met the inclusion criteria. Discrepancies were solved by consensus and through discussion with a third reviewer (RB or BHH).

### 2.4 Data Collection Process

Two authors (DDS and TB) extracted data in an adjusted template in Covidence from SRs as follows: authors, year, country, language, literature search date, registered study protocol (yes/no), databases and additional searches, aim of the study, study funding sources, and possible conflicts of interest for study authors. In addition, information on the total number of included primary studies for each SR and the number of relevant studies for each SR (the primary studies that pertained to the present review aim) was collected. For the relevant studies, the authors identified the type of study design and the methodological quality. For some SRs, the Grading of Recommendations, Assessment, Development and Evaluations (GRADE) was reported [21].

Other information extracted were the number of participants, sex, and age, the diagnostic system (DC/TMD, RDC/TMD, other), diagnoses, intervention, comparison group, results, and conclusions. One senior reviewer (RB or BHH) resolved any conflicts.

### 2.5 Risk of Bias Assessment

The AMSTAR‐2 [22] was used independently by two authors (DDS and TB) to assess the risk of bias of the SRs included in the study. AMSTAR‐2 comprises 16 items, each of which could be answered ‘Yes’, ‘Partial Yes’, ‘No’ (or not applicable). Of the 16 items, 7 are critical items, including prior protocol, comprehensive literature search, justification of excluding studies, assessment of RoB for individual studies, appropriate meta‐analytic methods, consideration of RoB in results, and impact of publication bias. On the basis of critical items, the overall methodological quality of the SR was rated as follows: high, moderate, low, and critically low.

Any disparity was resolved by consensus with a third reviewer (RB or BHH).

## 3 Results

The literature search identified 2390 articles, and after removal of duplicates 1740 records were imported in Covidence for title and abstract screening. In total, 399 were considered suitable for full‐text assessment, of which 151 were excluded. The main reason for the exclusion was not dealing with OS therapy (*n* = 227 studies). Full list of excluded studies at full‐text assessment level is reported in Table 1 and in Appendix S2.

**TABLE 1:** Full text not found (*n* = 7) and main reasons for exclusion at full‐text level (*n* = 378)^a^.

Main reason for exclusion	Study
Full text not found (*n* = 7)	(Ateneo Argentino de, 2007; Del Vecchio et al., 2023; Milutka et al., 2023; Morales, 1992; Sánchez Torres and Toranzo Fernández, 1994; Sandoval et al., 1997; Saranya et al., 2019)
Not occlusal splint (*n* = 227)	(Abdel Shaheed et al., 2019; Abdul et al., 2023; Aggarwal et al., 2019; Ahmad et al., 2021; Ahmadi 2020; Al‐Baghdadi et al., 2014; Al‐Hamed et al., 2021; Al‐Moraissi 2015; Al‐Moraissi 2015; Al‐Moraissi et al., 2022; Al‐Moraissi et al., 2017; Alsarhan et al., 2022; Alves et al., 2013; Alves et al., 2010; Ansari et al., 2022; Arbildo‐Vega et al., 2021; Argueta‐Figueroa et al., 2022; Armijo‐Olivo et al., 2016; Askar et al., 2021; Asquini et al., 2022; Assis et al., 2012; Ávila‐Curiel et al., 2020; Awan et al., 2019; Bahgat et al., 2023; Bankersen et al., 2021; Barry et al., 2022; Batista et al., 2022; Batista et al., 2022; Bavarian et al., 2021; Bou et al., 2022; Bouchard et al., 2017; Bousnaki et al., 2018; Brantingham et al., 2013; Brighenti et al., 2023; Brochado et al., 2019; Calixtre et al., 2015; Cascos‐Romero et al., 2009; Castaño‐Joaqui et al., 2017; Chang et al., 2014; Chen et al., 2015; Cho et al., 2010; Christidis et al., 2019; Chęciński et al., 2022; Chęciński et al., 2022; Chęciński et al., 2023; da Silva et al., 2023; da Silva et al., 2023; da Silviera et al., 2022; Dall et al., 2013; Davoudi et al., 2018; de Castro‐Carletti et al., 2023; de Freitas et al., 2013; de la Barra Ortiz et al., 2023; De la Torre Canales et al., 2019; de Melo et al., 2020; de Oliveira‐Souza et al., 2023; de Souza et al., 2012; Derwich et al., 2023; Derwich et al., 2021; Di Francesco et al., 2022; Dickerson et al., 2017; Dinsdale et al., 2022; El‐Kahky et al., 2022; Emérito et al., 2022; Ernst et al., 1999; Farshidfar et al., 2023; Feng et al., 2019; Fernandes et al., 2017; Fernandes et al., 2015; Fernández‐Hernández et al., 2017; Fernandez‐Vial et al., 2021; Ferreira et al., 2018; Ferreira et al., 2019; Ferreira et al., 2021; Ferrillo et al., 2022; Ferrillo et al., 2022; Fertout et al., 2022; Fisher et al., 2018; Florjanski et al., 2019; Forsell et al., 2004; Furlan et al., 2015; Goiato et al., 2016; Goker et al., 2021; González‐Sánchez et al., 2023; Gopi et al., 2021; Grossman et al., 2022; Guarda‐Nardini et al., 2021; Gutiérrez et al., 2022; Haddad et al., 2023; Haigler et al., 2018; Hanna et al., 2021; Herpich et al., 2015; Herranz‐Aparicio et al., 2013; Herrera‐Valencia et al., 2020; Herrero Babiloni et al., 2018; Hu et al., 2023; Hu et al., 2023; Huggins et al., 2012; Häggman‐Henrikson et al., 2017; Idáñez‐Robles et al., 2023; Januzzi et al., 2013; Jara Armijos et al., 2020; Jedel et al., 2003; Jing et al., 2021; Jung et al., 2011; Kim et al., 2023; Kotiranta et al., 2014; Kulkarni et al., 2020; La Touche et al., 2010; La Touche et al., 2010; La Touche et al., La Touche et al., 2020; La Touche et al., 2022; Lam et al., 2023; Law et al., 2015; Lee et al., 2023; Leite et al., 2009; Leung et al., 2020; Li et al., 2012; Li et al., 2021; Li et al., 2022; Li et al., 2023; Liapaki et al., 2021; Liberato et al., 2023; Lima et al., 2013; List et al., 2003; Liu et al., 2021; Liu et al., 2021; Liu et al., 2018; Liu et al., 2012; Machado et al., 2020; Machado et al., 2013; Machado et al., 2012; Machado et al., 2012; Machado et al., 2012; Machado et al., 2018; Maia et al., 2012; Manfredini et al., 2010; Manfredini et al., 2017; Marlière et al., 2023; Martin et al., 2011; Martin et al., 2012; Martins et al., 2016; Máximo et al., 2022; McNeely et al., 2006; Medlicott et al., 2006; Melis et al., 2012; Melis et al., 2022; Melo et al., 2018; Melo et al., 2018; Mena et al., 2020; Menéndez‐Torre et al., 2023; Moldez et al., 2018; Monteiro et al., 2020; Montinaro et al., 2022; Moon et al., 2013; Mujakperuo et al., 2010; Munguia et al., 2018; Müggenborg et al., 2023; Nagori et al., 2021; Nagori et al., 2018; Nandhini et al., 2018; Nimonkar et al., 2022; Ortiz et al., 2022; Paço et al., 2016; Park et al., 2023; Patel et al., 2019; Peixoto et al., 2019; Penlington et al., 2022; Petrucci et al., 2011; Pimentel et al., 2018; Pimentel et al., 2021; Pliavga et al., 2022; Porporatti et al., 2019; Prado‐Posada et al., 2020; Ramos‐Herrada et al., 2022; Randhawa et al., 2016; Ren et al., 2022; Rodhen et al., 2022; Roldán‐Barraza et al., 2014; Rosted 1998; Ruiz‐Romero et al., 2022; Sàbado‐Bundó et al., 2021; Sakalys et al., 2020; Salami et al., 2023; Sales et al., 2020; Santos et al., 2021; Saraiva et al., 2019; Sassi et al., 2018; Schindler et al., 2007; Senye et al., 2012; Serrano‐Muñoz et al., 2023; Shimada et al., 2023; Shukla et al., 2016; Siewert‐Gutowska et al., 2023; Sit et al., 2021; Sobral et al., 2021; Sommer 2002; Song et al., 2018; Sposito et al., 2014; Story et al., 2016; Sung et al., 2021, Tengrungsun et al., 2012; Thambar et al., 2020; Thorpe et al., 2023; Torres‐Rosas et al., 2023; Tournavitis et al., 2023; Tsui et al., 2022; Tunér et al., 2019; Türp et al., 2007; Türp et al., 2007; Undabarrerna et al., 2020; Vallejo‐Rosero et al., 2019; van der Meer et al., 2020; Vidya et al., 2015; Vieira et al., 2023; Vier et al., 2019; Vos et al., 2013; Votrubec et al., 2022; Webb et al., 2016; Worasing et al., 2023; Wu et al., 2021; Xu et al., 2018; Xu et al., 2023; Zhang et al., 2015; Zhang et al., 2020; Zhang et al., 2023; Zwiri et al., 2020)
Not systematic review (*n* = 73)	(Agurto P et al., 2004; Albagieh et al., 2023; Algabri and Alqutaibi, 2017; Araneda et al., 2013; Armijo‐Olivo et al., 2015; Arribas‐Pascual et al., 2023; Asquini et al., 2021; Barbosa et al., 2018; Beecroft et al., 2021; Beyer et al., 2022; Butts et al., 2017; Calatrava Oramas, 2014; Calderon, 2008; Chalco Valdivia and López Flores, 2019; Chauvel‐Lebret et al., 2013; Chen et al., 2021; Craane et al., 2018; Dall et al., 2015; Dammling et al., 2022; Dommerholt et al., 2016; Dommerholt et al., 2017; Dziedzic et al., 2008; Elbarbary et al., 2022; Fakhry and Abd‐Elwahab Radi, 2018; Fassina et al., 2016; Fricton, 2006; Furlan, 2011; Gallardo Leyva et al., 2018; Gallego Duque and Hincapie Mora, 1988; Ganem et al., 2023; Goldberg et al., 2003; González Mendoza and Hernández Calva, 2007; Grossmann and Grossmann, 2011; Grossmann, 2012; Grossmann et al., 2012; Guarda‐Nardini et al., 2017; Hsing and Ilankovan, 2020; Iturriaga et al., 2017b; Kietrys et al., 2014; Leite et al., 2013; Li and Shi, 2011; Lima et al., 2004; List and Axelsson, 2010; Machado et al., 2014; Maluf et al., 2008; Mélou et al., 2023; Monje‐Gil et al., 2012; Moraes et al., 2013; Morales Trejo, 2003; Naik et al., 2014; Netto et al., 2007; Penlington et al., 2019; Quinelato et al., 2011; Raman et al., 2023; Reginster et al., 2007; Rinchuse and McMinn, 2006; Rizzatti‐Barbosa and Albergaria‐Barbosa, 2018; Santander et al., 2011; Santonocito et al., 2023; Sembronio et al., 2021; Shaffer et al., 2014; Silva and Grillo, 2011; Singh et al., 2017; Soviero et al., 1997; Torres et al., 2020; Valladares‐Neto et al., 2014; Vasconcelos et al., 2006; Vasconcelos et al., 2008; Velasco and Salazar de Plaza, 2003; Vergara Muñoz, 1996; Wen et al., 2023; Weyant, 2006; Wiffen, 2011)
Not TMD population (*n* = 28)	(Abdul and Minervini, 2023; Almășan et al., 2023; Amat and Tran Lu, 2023; Azzopardi and Whitaker, 2010; Barry et al., 2021; Chęciński et al., 2023; Desai et al., 2022; Gonzalez et al., 2023; Harrison and Jones, 2013; Javed et al., 2021; Jazayeri et al., 2023; Katsnelson et al., 2012; Manrriquez et al., 2021; McQuay et al., 1995; Merz et al., 2022; Minervini et al., 2023a; Mittal et al., 2019; Monteiro et al., 2021; Moussa et al., 2023; Niezen et al., 2023; Olate et al., 2023; Priyank et al., 2023; Singh, 2013; Sterniczuk et al., 2022; Stoustrup et al., 2013; Te Veldhuis et al., 2017; Yaseen et al., 2021; Zheng et al., 2023)
Wrong outcome measure (*n* = 20)	(Akinbami, 2011; Al‐Baghdadi et al., 2014; Bach et al., 2022; Bermell‐Baviera et al., 2016; de Almeida et al., 2016; De Meurechy and Mommaerts, 2018; Ferrillo et al., 2022; Furlan, 2015; Iturriaga et al., 2017a; Luo et al., 2023; Melo et al., 2017; Nowak et al., 2021; Owen et al., 2022; Palmer et al., 2023; Prechel et al., 2018; Serrera‐Figallo et al., 2020; Sharma et al., 2022; Tesch et al., 2021; Tocaciu et al., 2019; Varedi and Bohluli, 2015)
Not a treatment study (*n* = 12)	(Al‐Riyami et al., 2009; Alam et al., 2023; Bessa‐Nogueira et al., 2008; Coronel‐Zubiate et al., 2022; Ding et al., 2022; Farook and Dudley, 2023; Ivorra‐Carbonell et al., 2016; Jamali et al., 2022; Kamińska et al., 2020; Langaliya et al., 2023; Mehraban et al., 2020; Minervini et al., 2023b)
Review updated or withdrawn (*n* = 8)	(Al‐Ani et al., 2016; Guo et al., 2015; Kim et al., 2018; Koh and Robinson, 2016; Luther et al., 2016; Rigon et al., 2015; Riley et al., 2020; Shi et al., 2013)
Language (*n* = 4)	(Alajbeg et al., 2015; Pan et al., 2012; van Selms et al., 2009; Zhang et al., 2008)
Not a full‐text publication (*n* = 3)	(Al‐Moraissi et al., 2022; Brignardello‐Petersen, 2019; Gadhia and Walmsley, 2009)
Others (*n* = 3)	(Naeije et al., 2013; Pereira and Hassan, 2022; Singh et al., 2022)

^a^ Full reference list is provided in Appendix S2.

In total, 248 SRs were included and categorised into five groups based on the main therapy evaluated: self‐management therapy, physiotherapy, occlusal splint therapy, maxillofacial surgery and orthodontics, pharmacological treatments and multiple/multimodal therapies.

**FIGURE 1:**
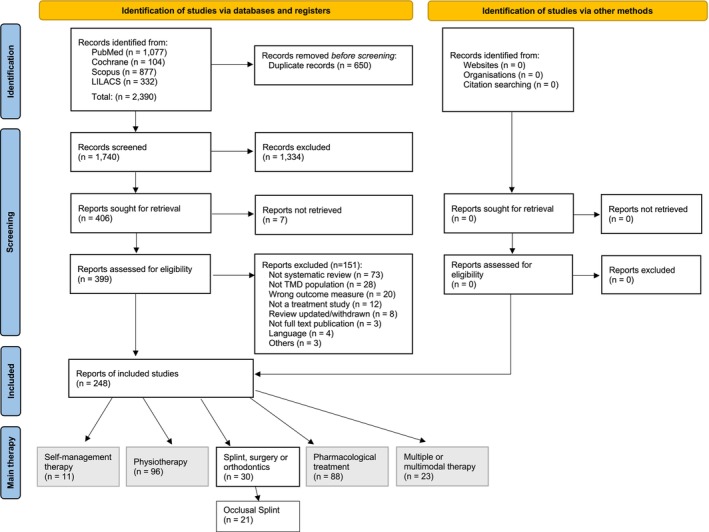
PRISMA flowchart of included and excluded studies.

Considering the purpose of the present umbrella review, 21 SRs were finally included as they focused on OS therapy [23, 24, 25, 26, 27, 28, 29, 30, 31, 32, 33, 34, 35, 36, 37, 38, 39, 40, 41, 42, 43]. The selection process is given in Figure 1.

Summarised data extracted from the 21 SRs are presented in Table 2. Nineteen of the studies were written in English and 2 in Spanish language [34, 38].

**TABLE 2:** Overview of included SRs.

First author Year Country	Aim	Included studies Nr. of relevant studies Study design	Study population Nr. of participants Diagnostic system	Intervention	Comparison	Outcome measures	Follow‐up	Results
Forssell [41] 1999 Finland	To investigate whether studies are in agreement with current clinical practices on occlusal treatments.	18 RCT	N/R N/R	Intraoral appliances; Occlusal adjustment	Behavioural therapy/Cognitive Behavioural therapy; Jaw excercises; Acupuncture; Electrophysical (TENS, laser, ultrasound, biofeedback); Occlusal adjustment; Placebo; Waiting list; No treatment	Pain intensity; Quality of Life	4–52 weeks	Splint therapy was found superior to 3 interventions and comparable to 12 control treatments, and superior or comparable to 4 passive controls, respectively. Occlusal adjustment was found comparable to 2 and inferior to one control treatment and comparable to passive control in one study. The use of occlusal splints may be of some benefit in the treatment of TMD. Evidence for the use of occlusal adjustment is lacking.
Türp [24] 2004 Switzerland	To assess if the use of a full‐coverage hard acrylic occlusal appliance lead to a significant decrease of symptoms. Is symptom improvement achieved with a stabilisation splint more pronounced than the success attained with other forms of management (including placebo) or no treatment?	9 RCT	701 RDC/TMD, Patient history and/or clinical findings	Intraoral appliances	Jaw excercises; Acupuncture; Intraoral appliances; Occlusal adjustment; Placebo; No treatment; Other: Conservative treatment	MMO; Pain; Quality of Life; Others	6 weeks–1 year and 27 weeks	Based on the currently best available evidence it appears that most patients with masticatory muscle pain are helped by the incorporation of a OS. Nevertheless, evidence is equivocal if improvement of pain symptoms after incorporation of the intraoral appliance is caused by a specific effect of the appliance.
Al‐Ani 2005 UK [23]	To determine the effectiveness of stabilisation splint therapy in reducing symptoms in patients with myofascial pain.	12 RCT	496 Reported own diagnostic criteria	Intraoral appliances	Behavioural therapy/Cognitive Behavioural therapy; Jaw excercises; Acupuncture; Electrophysical (TENS, laser, ultrasound, biofeedback); Intraoral appliances; No treatment	MMO; Pain; Quality of Life	4 weeks–6 months	There is insufficient evidence either for or against the use of OS therapy over other active interventions for the treatment of temporomandibular myofascial pain.
Stapelmann [28] 2008 Switzerland	To appraise the currently available evidence regarding the efficacy and safety of the NTI‐tss splint	3 RCT	76 RDC/TMD, patient history + clinical examination, Helkimo's Index	Intraoral appliances	Information/Education; Intraoral appliances	MMO; Pain; Adverse effects; Others	3–6 months	Evidence from RCTs suggests that the NTI‐tss device may be successfully used for the management of bruxism and TMDs. However, to avoid potential unwanted effects, it should be chosen only if certain a patient will be compliant with follow‐up appointments.
Fricton [39] 2010 United States	To conduct a SR of RCTs that have assessed the efficacy of intraoral orthopaedic appliances to reduce pain in patients with TMDs	44 RCT	2218 N/R	Intraoral appliances	Information/Education; Behavioural therapy/Cognitive Behavioural therapy; Jaw excercises; Acupuncture; Electrophysical (TENS, laser, ultrasound, biofeedback); Pharmacological (drugs); Occlusal adjustment; Placebo; No treatment	MMO; Pain	7 days–1 year	In the first meta‐analysis a hard stabilisation appliance was found to improve TMJD pain compared to non‐occluding appliance. In the second meta‐ analysis, a hard stabilisation appliance was found to improve TMJD pain compared to no‐treatment controls.
Ebrahim [42] 2012 Canada	To evaluate whether splint therapy is more effective than minimal or no treatment in patients with TMD.	11 RCT	455 N/R	Intraoral appliances	No treatment; Other: Minimal treatment	Pain; Quality of Life; Others	6 weeks–12 months	Moderate‐quality evidence suggests that splint therapy reduced pain in the TMJ area. Low to very low quality of evidence showed no significant differences between the splint therapy and control groups in terms of quality of life or depression. None of the trial reports described effect on function.
Pficer [25] 2017 Serbia	To determine short‐ and long‐term effects of stabilisation splint (SS) in treatment of TMDs, and to identify factors influencing its efficacy.	33 RCT	1779 RDC/TMD, clinical examination, criteria by De Leeuw, International Headache Society, radiography	Intraoral appliances	Information/Education; Behavioural therapy/Cognitive Behavioural therapy; Jaw excercises; Intraoral appliances; Placebo; No treatment; Other: Physical therapy	MMO; Pain	1month–12month	OS presented short term benefits for patients with TMDs. In long term follow up, the effect is equalised with other therapeutic modalities.
Nagori [37] 2019 India	To assess the efficacy of splint therapy in improving outcomes after arthrocentesis for the management of TMJD.	3 RCT 2 Case Control Study Prospective 1 Case Control Study Retrospective	235 N/R	Intraoral appliances; Surgery	Surgery	MMO; Pain	1 month–1 year	There was no statistical significant difference in pain reduction with or without the use of splint after arthrocentesis at 1 month and 6 months. Similarly, no difference was seen in improvement in MMO at 1 month. Within the limitation of this review, there is some evidence that splint therapy may not improve outcomes after arthrocentesis.
Fouda [40] 2020 Egypt	To determine the efficacy of oral splints in reducing the intensity of pain in patients with TMD disfunction in both short and long‐term treatment durations	22 RCT	2068 RDC/TMD	Intraoral appliances	Information/Education; Behavioural therapy/Cognitive Behavioural therapy; Jaw excercises; Electrophysical (TENS, laser, ultrasound, biofeedback); Pharmacological (drugs); Intraoral appliances; No treatment	MMO; Pain	1 month–1 year	A meta‐analysis of short‐term studies up to 3 months revealed no significant difference between the study groups. However, long‐term studies exhibited a significant difference in pain reduction in favour of the control group. Oral splints are not effective in reducing pain intensity or improving function in patients with TMJ dysfunction.
Riley [35] 2020 UK	To evaluate the clinical‐effectiveness of oral splints for patients with TMD or bruxism for the primary outcomes: pain (TMD) and tooth wear (bruxism).	35 RCT	2319 RDC/TMD, DC/TMD, clinical features, Helkimo index, MRI, arthrography	Intraoral appliances	Information/Education; Manual Therapy (Joint mobilisation, manipulation…); Jaw excercises; Electrophysical (TENS, laser, ultrasound, biofeedback); Joint injection; No treatment; Other	MMO; Pain; Adverse effects; Quality of Life	Up to 12 months	There was no evidence that splints reduced pain. There was no evidence that any other outcomes improved when using splints. There was no evidence of adverse events associated with splints, but reporting was poor regarding this outcome. No trials measured tooth wear in patients with bruxism. The very low‐quality evidence identified did not demonstrate that splints reduced pain in TMDs as a group of conditions. There is insufficient evidence to determine whether or not splints reduce tooth wear in patients with bruxism.
Blanchard [43] 2021 UK	The aim of this systematic review was to analyse the effectiveness of an oral splint for the treatment of muscular TMD in children and understand the significance of this treatment intervention in reducing pain.	2 RCT	186 RDC/TMD	Information/Education; Behavioural therapy/Cognitive Behavioural therapy; Intraoral appliances	Information/Education; Behavioural therapy/Cognitive Behavioural therapy; No treatment	MMO; Pain; Others	3–6 months	There is insufficient reliable evidence for the use of an oral splint for the treatment of myofascial pain in growing patients.
Hidalgo Ordoñez [38] 2021 Ecuador	To test whether occlusal splints control pain related to temporomandibular disorders.	8 Case Control Study Prospective 5 Cohort Study Prospective	N/R N/R	Intraoral appliances	Information/Education; Jaw excercises; Placebo; No treatment	MMO; Pain; Adverse effects	1 to 36 months	OS are efficient devices that meet the goal of controlling symptomatology in patients with TMDs.
Zhang [32] 2021 China	To compare the effects of exercise therapy and occlusal splint therapy on pain and mobility in individuals with painful TMD.	6 RCT	498 RDC/TMD	Intraoral appliances	Jaw exercises	MMO; Pain; Others	N/R	The results revealed that exercise therapy was not superior to OS therapy for pain reduction in patients with painful TMD. The effectiveness of OS therapy and exercise therapy was found to be equivalent in the MMO range, right laterotrusion, left laterotrusion, and protrusion for painful TMD patients. No high‐quality evidence was found to distinguish the clinical effectiveness between OS therapy and exercise therapy for painful TMD patients.
Freire‐Nieto [34] 2022 Spain	To search for clinical findings in quality studies on the effectiveness of occlusal splint therapy.	21 studies N/R the type	1370 RDC/TMD	Intraoral appliances	Information/Education; Behavioural therapy/Cognitive Behavioural therapy; Jaw exercises; Electrophysical (TENS, laser, ultrasound, biofeedback); Joint Injection; Placebo; No treatment Other: Ozone therapy	MMO; Pain; Others	1 year	OS effectively reduce painful symptoms in patients with TMDs, both in muscular and joint pathologies.
Honnef [26] 2022 Brazil	To assess effects of stabilisation splints on signs and symptoms of temporomandibular disorders of muscular origin compared to other treatments.	10 RCT	369 RDC/TMD, DC/TMD, AAOP and ICOP	Intraoral appliances	Information/Education; Manual Therapy (Joint mobilisation, manipulation…); Electrophysical (TENS, laser, ultrasound, biofeedback); Pharmacological (botox injection); Intraoral appliances; Placebo; Other: Trigger point local anaesthetic injection	MMO; Pain; Others	1 week −6 months	OS was reported to be as effective as other treatments on analysed outcomes. The certainty of evidence was very low for all outcomes. Positive effect on signs and symptoms of TMDs of muscular origin, when managed with OS, could not be confirmed or refuted.
Muresanu [31] 2022 Romania	To assess the therapeutic efficacy of computer‐assisted or digitally constructed occlusal splints in comparison to conventional splint treatment for temporomandibular disorders or bruxism.	4 RCT 3 Case Control Study Prospective	244 RDC/TMD DC/TMD and others	Intraoral appliances	Intraoral appliances	MMO; Pain; Others	3 weeks ‐ 9 months	Computer aided design OS provide equivalent outcomes to traditional splints.
Bhattacharjee [27] 2023 India	To evaluate the efficacy of arthrocentesis procedure in comparison with stabilization splints used for disc displacement disorders without reduction.	3 RCT 1 Case Control Prospective 1 Case Control Retrospective	379 RDC/TMD, clinical findings + MRI	Joint injection; Intraoral appliances	Manual Therapy (Joint mobilisation, manipulation…); Jaw excercises; Electrophysical (TENS, laser, ultrasound, biofeedback); Pharmacological (drugs); Intraoral appliances	MMO; Pain	1 month −12 months	Overall, results supported the rationale of using arthrocentesis in patients with DDwoR.
Denardin [33] 2023 Brazil	To determine the best disocclusion guidance in occlusal splints to manage and treat temporomandibular disorder and sleep bruxism.	4 RCT 4 Case Control Prospective 6 Cohort Study Prospective	573 RDC/TMD, DC/TMD, AAOP	Intraoral appliances	Intraoral appliances; Placebo; No treatment	MMO; Pain; Others	1 week to 6 months	It is suggested there is not enough evidence to support that there are any specific kind of guidance responsible for improving evaluated outcomes on TMDs.
Dhar [29] 2023 India	To assess the efficacy of anterior repositioning splints for ddwr and ddwor.	3 RCT 3 Case Control Prospective 3 Cohort Study Prospective 3 Cohort Study Retrospective	814 N/R	Intraoral appliances	Other: Not reported	Pain; Quality of Life; Others	4–12 months	The results suggested that both DDwR and DDwoR were effective in improving TMJ symptoms in patients who received ARS therapy.
Kelemen [30] 2023 Hungary	To compare the efficacy of combination therapy (splint therapy along with physiotherapy, manual therapy, and counselling) with physiotherapy, manual therapy, and counselling alone.	7 RCT	578 RDC/TMD	Information/Education; Behavioural therapy/Cognitive Behavioural therapy; Manual Therapy; Jaw exercises; Intraoral appliances	Information/Education; Behavioural therapy/Cognitive Behavioural therapy; Manual Therapy (Joint mobilisation, manipulation…); Jaw excercises; Pharmacological (drugs)	MMO; Pain; Quality of Life	1 month	Combination therapy compared to physiotherapy, manual therapy, and counselling alone due to the marginal differences between the baseline and 1‐month values could not confirm the efficacy of combination therapy.
Orzeszek [36] 2023 Poland	To examine the existing original studies to determine the effectiveness of OSs in the management of orofacial myalgia and myofascial pain (MP) in comparison with no treatment or other interventions.	13 RCT	589 RDC/TMD and DC/TMD	Intraoral appliances	Information/Education; Behavioural therapy/Cognitive Behavioural therapy; Jaw excercises; Acupuncture; Electrophysical (TENS, laser, ultrasound, biofeedback); Other: Myofunctional therapy, Kinesio Taping	MMO; Pain; Others	2 weeks ‐ 12 months	Data to draw definitive conclusions on the effectiveness of OS compared with no treatment or other intervention for the treatment of orofacial myalgia and MP were insufficient.

Abbreviations: AAOP, American Academy of Orofacial Pain; DC/TMD, Diagnostic Criteria for Temporomandibular Disorders; DDwoR, Disc Displacement without Reduction; DDwR, Disc Displacement with Reduction; ICOP, International Classification of Orofacial Pain; MMO, Maximum Mouth Opening; MRI, Magnetic Resonance Imaging; N/R, Not Reported; OS, Occlusal Splint; RCT, Randomised Controlled Trial; RDC/TMD, Research Diagnostic Criteria for Temporomandibular Disorders; TMD, Temporomandibular Disorders.

The articles were written in various countries: UK (3), India (3), Brazil (2), Switzerland (2), Poland (1), Spain (1), Romania (1), Hungary (1), China (1), Serbia (1), Ecuador (1), USA (1), Egypt (1), Finland (1), Canada (1).

The number of primary studies included in each SR ranged between 2 and 44.

Most of the SRs included only RCTs, but 6 SRs [27, 29, 33, 34, 37, 38] also included prospective and retrospective case–control or cohort studies. One SR [34] did not report the type of primary studies included. Six SRs had the protocol registered on PROSPERO [26, 30, 31, 32, 33, 43] and 10 SRs were integrated with a meta‐analysis [25, 27, 29, 30, 32, 35, 37, 39, 40, 42]. In 6 studies, GRADE was reported [25, 26, 30, 33, 35, 42].

The total number of subjects included in each review varied from 76 to 2319, with two SRs not reporting the number [38, 41]. The majority of the studies focused on different TMD diagnoses, while three SRs [27, 29, 37] focused only on TMJ pain and five SRs [24, 26, 30, 36, 43] only on muscle pain.

The diagnostic criteria that have been more frequently used were the RDC/TMD and the DC/TMD [1, 44]. Six studies [29, 37, 38, 39, 41, 42] did not report the diagnostic system adopted for patient selection, and one study [23] used its own diagnostic criteria.

The main types of OS used were the Michigan splint, Anterior Repositioning Splint, and Nociceptive Trigeminal Inhibition – tension suppression system (NTI‐tss). One SR [31] compared traditionally made OS with computer‐designed OS.

The follow‐up period ranged from 1 month to 36 months, with one SR [32] not reporting this information.

### 3.1 Clinical Findings

#### 3.1.1 Occlusal Splint in Adolescents

Blanchard et al. [43] examined the use of OS in growing patients and concluded that it is not an effective treatment for myofascial pain in this age group. There was no statistically significant difference in Maximum Mouth Opening (MMO) across the different interventions received and the control group.

#### 3.1.2 Occlusal Splint vs. Jaw Exercises

In the SR by Zhang and colleagues [32], exercise therapy did not show better results than OS therapy in reducing pain for patients with painful TMD. The effectiveness of OS therapy and exercise therapy was determined to be similar in terms of the MMO range, right and left lateral movement, and protrusion for patients with painful TMDs.

#### 3.1.3 Various Occlusal Splint Designs

Stapellmann and co‐workers [28] analysed the NTI‐tss device compared with other designs of OS, with evidence from RCTs suggesting that the NTI‐tss device may be effectively used for managing TMDs.

Muresanu and colleagues [31] compared computer‐aided design OS with traditional splints and concluded that they produce similar outcomes.

In the study conducted by Denardin et al. [33], various OS designs, including canine guidance, bilateral balanced occlusion, anterior guidance, and molar guidance, were examined. The authors concluded that there is not enough evidence to support any benefits on TMD from a specific kind of guidance.

Dhar and co‐workers [29] suggested that both in patients with disc displacement with reduction and disc displacement without reduction, anterior repositioning splint was effective in improving TMJ symptoms. The efficacy levels ranged from 71% to 83% for disc displacement with reduction and from 50% to 95% for disc displacement without reduction.

#### 3.1.4 Occlusal Splint Versus No Treatment

Ebrahim et al. [42] found moderate‐quality evidence that OS therapy reduced pain in the TMJ area. However, low to very low quality of evidence showed no significant differences between the OS therapy and no‐treatment controls in terms of quality of life.

#### 3.1.5 Occlusal Splint and Arthrocentesis vs. Arthrocentesis Only

According to Nagori et al. [37], there was no statistically significant difference in pain reduction with or without the use of a splint after arthrocentesis at 1 month. Similarly, no difference was seen in improvement in MMO at 1 month and at 6 months.

#### 3.1.6 Arthrocentesis vs. Occlusal Splint

In the study by Bhattacharjee and colleagues [27], arthrocentesis appeared to be more effective than OS and other non‐invasive approaches in improving MMO and reducing pain levels in patients with disc displacement without reduction.

#### 3.1.7 Occlusal Splint vs. Multiple Conservative Treatments

In 12 SRs, primary studies were included if they compared OS treatment with a variety of non‐invasive approaches for TMD management. These comparisons included behavioural therapy, jaw exercises, acupuncture, electrophysical treatments (such as TENS, laser, ultrasound and biofeedback), occlusal adjustment, placebo, waiting list, and no treatment.

According to four SRs [34, 38, 39, 41], OS therapy was effective for treating TMDs. Forssell et al. [41] compared intraoral appliances and occlusal adjustment with other interventions and found OS therapy to be superior in reducing pain and improving the quality of life. In two meta‐analyses by Fricton et al. [39], it was found that a hard stabilisation appliance improved TMJ pain compared to non‐occluding appliances and no‐treatment controls. Additionally, both Hidalgo Ordoñez et al. [38] and Freire‐Nieto et al. [34] concluded that OS, compared to different conservative treatments, effectively reduces painful symptoms in patients with TMDs, including both muscular and joint pathologies.

On the other hand, five SRs reported limited evidence to support or dismiss the use of OS therapy over other active interventions for TMD treatment [23, 24, 25, 26, 30]. In particular, Türp et al. [24] found positive results when comparing intraoral appliances with other treatments, but the authors claimed that it is uncertain whether the reported pain reduction was specifically due to the effect of the OS. The SR by Al‐Ani et al. [23] suggested that OS therapy may be beneficial in reducing pain severity at rest and on palpation and symptoms of depression compared to no treatment, but the evidence was insufficient either for or against the OS therapy. Furthermore, the authors found no statistically significant difference in the increase in MMO between groups receiving OS or a non‐occluding splint. Pficer et al. [25] reported that in the short term, OS therapy had a positive overall effect on pain reduction, muscle tenderness, and mouth opening, but the quality of the evidence was low. In the long term, the effects of OS therapy were not superior to other therapeutic modalities. Honnef et al. [26] found OS to be equally as effective as other treatments in improving TMD symptoms of muscular origin for the analysed outcomes (pressure pain threshold, pain during chewing, mouth opening, spontaneous pain intensity and by palpation). Kelemen et al. [30] compared the efficacy of combined therapy (splint therapy with physiotherapy, manual therapy, and counselling) with physiotherapy, manual therapy and counselling alone and found minimal differences between the two approaches.

According to three SRs, OS was not more effective than other treatment modalities for TMD treatment [35, 36, 40]. In particular, the meta‐analysis by Fouda et al. [40] showed that short‐term primary studies found no significant difference between study groups, while long‐term results did show a significant difference in favour of the control group in reducing pain. The study also supported that OS therapy was not effective in reducing pain intensity or improving function in patients with TMJ dysfunction. Riley et al. [35] showed no evidence that OS reduced pain or improved any other outcomes for TMDs. Finally, in the study by Orzeszek and colleagues [36], data were not sufficient to draw definitive conclusions on the effectiveness of OS for the treatment of orofacial myalgia and myofascial pain compared with no treatment or other interventions.

### 3.2 Methodological Quality of Included Reviews

The methodological quality of the included reviews as assessed with the AMSTAR‐2 ranged from critically low (5 studies), to low (4 studies), moderate (8 studies), and high (4 studies). The most common critical weakness in the included reviews was the absence of clearly a prior established review methods, any significant deviations from the protocol, the lack of assessment of RoB in individual primary studies, and the adoption of non‐standardised tools to assess the RoB in individual primary studies. (Table 3).

**TABLE 3:** Quality assessment of included SRs with AMSTAR‐2, presence of a GRADE assessment, and used risk of bias tool for the assessment.

Study	AMSTAR 2																GRADE	Quality assessment
	RQ, PICOS and protocol			Search, selection and extraction					Risk of Bias, Statistical Method & Analysis									
	1	2	3	4	5	6	7	8	9	10	11	12	13	14	15	16		
Orzeszek 2023	Y	PY	N	PY	Y	N	N	PY	Y	N	N/A	N/A	Y	Y	N	Y	No	RoB 2
Kelemen 2023	Y	Y	N	PY	Y	Y	N	PY	Y	N	Y	N	Y	Y	N	Y	Yes	RoB 2
Dhar 2023	Y	PY	N	PY	Y	Y	N	N	Y	N	N	N	N	Y	N	Y	No	RoB 2
Denardin 2023	Y	Y	N	Y	Y	Y	Y	PY	Y	N	N/A	N/A	Y	Y	N	Y	Yes	Joanna Briggs Institute of Critical Appraisal Tools
Bhattacharjee 2023	Y	PY	N	PY	Y	Y	Y	PY	Y	N	Y	N	N	Y	N	Y	No	RoB 2; Newcastle‐Ottawa Scale
Muresanu 2022	Y	Y	N	PY	Y	Y	N	PY	Y	N	N/A	N/A	Y	N	N	Y	No	RoB 2; ROBINS‐I
Honnef 2022	Y	Y	N	Y	Y	Y	Y	PY	Y	N	N/A	N/A	Y	Y	N	N	Yes	RoB 2
Freire‐Nieto 2022	N	PY	N	PY	Y	Y	N	PY	N	N	N/A	N/A	N	N	N	N	No	—
Zhang 2021	Y	Y	N	PY	Y	Y	N	PY	PY	N	Y	N	Y	Y	N	Y	No	Pedro
Hidalgo Ordoñez 2021	N	N	N	N	N	N	N	PY	N	N	N/A	N/A	N	N	N	Y	No	Jadad scale
Blanchard 2021	Y	PY	N	PY	Y	Y	Y	PY	Y	N	N/A	N/A	Y	Y	N	Y	No	RoB 2
Riley 2020	Y	N	N	PY	Y	Y	N	Y	Y	Y	Y	Y	Y	Y	N	Y	Yes	RoB 2
Fouda 2020	Y	N	N	PY	N	N	N	PY	Y	N	Y	N	N	Y	Y	Y	No	RoB 2
Nagori 2019	Y	PY	N	PY	Y	Y	N	PY	N	N	Y	N	Y	Y	N	N	No	MOOSE, STROBE statement and PRISMA statement
Pficer 2017	Y	N	N	N	Y	Y	PY	PY	Y	N	Y	N	Y	Y	Y	Y	Yes	RoB 2 and Jadad scale
Ebrahim 2012	N	N	N	N	Y	Y	N	N	Y	N	Y	N	N	Y	N	N	Yes	Modified RoB
Fricton 2010	Y	PY	Y	PY	Y	Y	N	PY	Y	N	Y	N	Y	Y	Y	N	No	CONSORT criteria
Stapelmann 2008	N	N	N	N	N	N	N	PY	PY	N	N/A	N/A	N	N	N	Y	No	Jadad scale
Al Ani 2005	Y	N	N	N	Y	Y	N	PY	PY	N	Y	Y	Y	Y	N	N	No	—
Türp 2004	N	N	N	Y	N	N	N	PY	PY	N	N/A	N/A	Y	N	N	N	No	Jadad scale
Forssell 1999	N	N	N	PY	Y	N	Y	PY	PY	N	N/A	N/A	Y	Y	N	N	No	Quality scale by Antczak et al.

*Note:* Red stands for 'No', yellow for 'Partial yes', green for 'Yes' and grey for 'Not applicable'.

Abbreviations: PICOS, Population‐Intervention‐Comparator‐Outcome‐Study design; PY, Partial Yes; RoB 2, Risk of Bias 2 Tool; ROBINS‐I, Risk Of Bias In Non‐randomised Studies ‐ of Interventions; RQ, Research Question; Y, Yes.

## 4 Discussion

TMD is a complex condition that necessitates a holistic approach to treatment [45]. Typically, conservative treatment options are preferred as a first‐line approach due to their safety, cost‐effectiveness, and ability to be easily administered. Among those, OS is widely used worldwide by both general dentists and specialists [46]. Despite the wide use, the effectiveness of OS remains a topic of debate. Therefore, the present overview was conducted to summarise the results from the published multiple SRs and to provide readers, clinicians, and researchers with a single paper that encompasses all the current evidence.

Even if most SRs support the clinical utility of OS for pain relief and jaw function, the evidence fails to demonstrate that OS are significantly superior to other therapeutic modalities. This lack of clear superiority suggests that the efficacy of OS might be comparable to other conservative measures, although from clinical experience it can be speculated that it is an efficacious protective tool to prevent damage from dental grinding.

Notwithstanding, it is crucial to carefully consider individual patient factors when applying OS. As a matter of fact, despite being considered a conservative treatment option, OS is not without potential side effects. For example, irreversible occlusal changes can occur, thus requiring close monitoring and adjustments [47]. Furthermore, long‐term reliance on splints without addressing underlying issues may lead to suboptimal outcomes [48]. In the current overview, only 4 out of the 21 included SRs reported data on adverse effects and the description was generally limited, so these data do not allow a consistent meta‐analysis of the frequency or severity of these side effects. In the SR by Riley et al., it is explicitly stated that no adverse events were reported in the included studies [35]. Muresanu et al. [31] provided a summary table indicating only the presence or absence of side effects in primary studies without specifying their clinical nature. In the papers by Stapelmann & Türp and Fricton et al. adverse effects were discussed primarily in relation to anterior repositioning splints to highlight their risks compared to stabilisation appliances [28, 39]. These reviews mention rare but significant concerns of stabilisation splints such as an increase in clenching activity or unintended irreversible occlusal changes resulting from full‐time wear. These findings support the suggestion of wearing the stabilisation appliance only during the nighttime (or limited daytime use during short periods). Other reported side effects, including transient changes in salivation or dental tension, are short‐lasting and manageable through minor clinical adjustments. It is important that in future studies researchers should be encouraged to systematically report any unintended outcomes to improve the quality of future meta‐analyses. For clinicians, it could be useful to record the starting occlusion of patients and systematically check if there are some occlusal changes during OS treatment.

From the current comprehensive bibliographic research, 21 SRs were included. The included SRs compared OS with various treatments or compared different types of splint designs and found controversial and sometimes conflicting results. Among the included SRs, extreme variety was found in terms of criteria used for diagnosis, devices analysed/evaluated, target population and comparison with other interventions [49]. In particular, great heterogeneity in applied inclusion and exclusion criteria among SRs was found, introducing wide differences in the studied treatment protocol (duration of interventions, follow‐up periods and outcome measures). Therefore, this heterogeneity makes it challenging to directly compare the results and generalise the findings to broader populations.

For instance, although validated diagnostic criteria have been established for TMD assessment since more than 30 years (RDC/TMD, first, and DC/TMD, after) [1, 44] the majority of the SRs included studies in which TMDs were diagnosed with miscellaneous approaches. This could render the diagnostic strategies incomparable between studies and return invalid results. Moreover, it has been recognised that TMDs are heterogeneous conditions that might present painful and/or dysfunctional symptoms, each with distinct etiological factors and clinical manifestations. Therefore, merging different TMD diagnoses in the same SR may limit the external validity of the results.

In addition, combining studies that evaluate different splints used for different purposes can result in inaccurate conclusions. This mix of heterogeneous data makes it challenging to draw precise and clinically applicable guidelines.

In summary, our findings are consistent with the existing body of literature that emphasises the variability in the effectiveness of OS compared to other treatments and underlines the need for a more personalised approach to treat TMDs [49].

In the majority of SRs focused on splint therapy, this treatment was frequently presented and analysed as the primary intervention for various conditions. However, a closer examination of the primary studies included in this overview reveals a critical limitation: splint therapy was almost invariably combined with other therapeutic modalities, such as physiotherapy or behavioural interventions.

This prevalent combination of treatments poses a significant challenge for evaluating the ‘pure’ effect of any single treatment modality, including splint therapy. When splint therapy is not isolated as the only treatment in clinical trials, it becomes difficult to attribute observed outcomes directly to the splint itself. Instead, the results are likely influenced by the synergistic or cumulative effects of the accompanying treatments, leading to a potential overestimation or underestimation of the true effect of OS. Consequently, the current body of literature does not provide a clear assessment of splint therapy's effectiveness when used alone. This gap in the evidence base does not permit clinicians and researchers to determine the true therapeutic value of splint therapy, independently from other interventions. Therefore, the specific contribution of OS therapy to pain relief in TMD patients needs to be more clearly distinct from the effects of concomitant behavioural or physical therapies.

To address this issue, future research should aim to design studies that specifically isolate splint therapy from other treatments. By doing so, researchers can more accurately assess its individual efficacy and better inform clinical practice. Additionally, SRs and meta‐analyses should carefully consider the confounding effects of combined treatments and, where possible, stratify results to distinguish between studies that utilise splints as the only therapy and those that do not. This approach will lead to a more precise understanding of the role and effectiveness of splint therapy in clinical settings.

Specifically, future research should favour RCTs with multiple arms to compare OS as a monotherapy against both placebo and multiple treatments. This would allow for a clearer quantification of the ‘added value’ that the splint provides compared to other conservative therapies. Moreover, the use of meta‐regression in future SRs could help to identify how much of the clinical improvement is specifically attributable to the appliance itself, rather than to the multimodal protocol as a whole.

In our overview, most of the SRs included (8 SRs) have a moderate quality score according to AMSTAR‐2, but 9 SRs range between critically low and low quality. Future SRs should adopt a more rigorous approach in planning and conducting reviews, with greater adherence to predefined protocols and adequate assessment of risk of bias to improve overall methodological quality.

From the results of the included SRs, it can be stated that OS does not show a significant improvement in quality of life [30, 35], and adverse events have been poorly studied, with the exception of those associated with NTI‐tss use, including unintended occlusal changes [28].

As previously stated in the methods, it must be acknowledged that this overview includes Systematic Reviews (SRs) identified through a comprehensive literature search conducted up to the end of 2023. Consequently, SRs published after this date were not included and might present different results.

The high degree of heterogeneity observed in the SRs included significantly impacts the generalisability of our conclusions. This variability is due to the diverse designs and wear protocols of OS across trials. Such heterogeneity makes it challenging to assess which specific patient phenotypes benefit most from a given appliance. To address these limitations in future research, it is essential to move towards a more standardised framework. Specifically, the adoption of the DC/TMD should be mandatory to ensure consistency in patient selection. Furthermore, treatment protocols should clearly define the material, thickness and precise wear duration (e.g., nighttime vs. 24‐h use) of the splints. Standardising outcome measures – using validated scales for pain intensity and functional jaw movement – would allow for more robust meta‐analyses and provide clinicians with clearer, evidence‐based guidelines for everyday practice.

Based on the observation of existing literature, the following recommendations should be considered when designing future SRs on TMD:

– Study Design: Include only Randomised Controlled Trials (RCTs) as primary studies whenever possible
– Diagnostic Criteria: Utilise diagnoses based on DC/TMD protocols
– Subgroup Analysis: Conduct separate studies or analyses for articular versus muscular diagnoses
– Intervention Clarity: Provide a detailed description of each intervention.
– Outcome Measures: Select a limited number of relevant, standardised outcome measures.

## 5 Conclusions

The overview contributes valuable insights into the current state of research on OS therapy for TMD management by providing a comprehensive summary of the existing evidence from SRs. The findings emphasise a substantial variability in the literature regarding treatment protocols, design of the devices and target population, thus limiting the possibility to draw conclusions that are largely applicable to a TMD patient population.

Overall, most SRs report beneficial effects of OS therapy, but the isolated effect of OS therapy could not be established. Therefore, the adoption of this treatment modality should be considered in the light of its cost‐effectiveness and aligned with patients' preferences and needs.

## Author Contributions

All authors reviewed and commented on the manuscript. DDS: study selection, data extraction, risk of bias assessment, data analysis and interpretation, drafting the manuscript and approval of final version. AM: conceptualisation, critical review of the manuscript and approval of the final version. RB: conceptualisation, study selection, data extraction, risk of bias assessment data analysis, critical review of the manuscript and approval of the final version. TB: study selection, data extraction and risk of bias assessment. TL: critical review of the manuscript and approval of the final version. BHH: conceptualisation, study selection, data extraction, risk of bias assessment, data analysis and interpretation, critical review of the manuscript and approval of the final version.

## Funding

The authors have nothing to report.

## Conflicts of Interest

The authors declare no conflicts of interest.

## Supporting information


**Appendix S1:** Electronic search strategy and identified records on December 1st 2023.
**Appendix S2:** Reference list of studies for which the full text was not found (*n* = 7) and studies excluded during full‐text assessment (*n* = 378)

## Data Availability

The presented data from the systematic reviews and their primary studies are available through an electronic database search.
