# Supplementary material for: Occlusal Splint Therapy in the Management of Temporomandibular Disorders—Evidence from Systematic Reviews
Source: J Oral Rehabil. 2026 Apr 30;53(8):1562–79. doi: 10.1111/joor.70208 (PMC13358406; doi:10.1111/joor.70208)
Supplement: Supplementary file 1 — Appendix S1: Electronic search strategy and identified records on December 1st 2023. Appendix S2: Reference list of studies for which the full text was not found (n = 7) and studies excluded during full‐text assessment (n = 378) [file JOOR-53-1562-s001.docx]

| Databases and search strategy | | Records |
| --- | --- | --- |
| PubMed via NLM |  |  |
| 1. TMJD | |  |
| **((((facial[Title/Abstract] OR jaw[Title/Abstract] OR orofacial[Title/Abstract] OR craniofacial[Title/Abstract] OR trigem*[Title/Abstract]) AND (pain[Title/Abstract])) OR (TMD[Title/Abstract] OR TMJD[Title/Abstract] OR TMJ disorder[Title/Abstract] OR TMJ dysfunction[Title/Abstract] OR TMJ disease[Title/Abstract] OR TMJ syndrome[Title/Abstract] OR TMJ disorders[Title/Abstract] OR TMJ diseases[Title/Abstract] OR TMJ syndromes[Title/Abstract] OR TMJ dysfunctions[Title/Abstract] OR temporomandibular joint disorders[Title/Abstract] OR temporomandibular joint disorder[Title/Abstract] OR temporomandibular disorders[Title/Abstract] OR temporomandibular disorder[Title/Abstract] OR temporomandibular joint disease[Title/Abstract] OR temporomandibular joint diseases[Title/Abstract] OR temporomandibular joint dysfunction syndrome[Title/Abstract] OR temporomandibular joint syndrome[Title/Abstract] OR temporomandibular joint dysfunction syndromes[Title/Abstract] OR temporomandibular joint syndromes[Title/Abstract] OR temporomandibular joint dysfunction[Title/Abstract] OR temporomandibular joint dysfunctions[Title/Abstract] OR temporomandibular dysfunction[Title/Abstract] OR temporomandibular dysfunctions[Title/Abstract] OR Craniomandibular Disorders[Title/Abstract] OR Craniomandibular Disorder[Title/Abstract] OR Craniomandibular Dysfunction[Title/Abstract] OR Craniomandibular Dysfunctions[Title/Abstract])) OR ("Craniomandibular Disorders"[Mesh]))** | | 46,604 |
| 2. Systematic review Systematic [sb] | |  |
| (((systematic review[ti] OR systematic literature review[ti] OR systematic scoping review[ti] OR systematic narrative review[ti] OR systematic qualitative review[ti] OR systematic evidence review[ti] OR systematic quantitative review[ti] OR systematic meta-review[ti] OR systematic critical review[ti] OR systematic mixed studies review[ti] OR systematic mapping review[ti] OR systematic cochrane review[ti] OR systematic search and review[ti] OR systematic integrative review[ti]) NOT comment[pt] NOT (protocol[ti] OR protocols[ti])) NOT MEDLINE [subset]) OR (Cochrane Database Syst Rev[ta] AND review[pt]) OR systematic review[pt] | | 288,588 |
| 1 AND 2 | | \| 1,077 \| \| --- \| |

**Appendix 1**. Electronic search strategy and identified records on December 1^st^ 2023.

| Scopus via Elsevier |  |  |
| --- | --- | --- |
| 1. TMJD | |  |
| (TITLE-ABS-KEY ("craniomandibular disorder" OR "Craniomandibular Dysfunction" OR "temporomandibular joint disorder" OR "temporomandibular disorder" OR "temporomandibular joint disease" OR "temporomandibular joint dysfunction" OR "temporomandibular joint syndrome" OR "temporomandibular dysfunction" OR tmjd OR tmd OR "tmj disorder" OR "TMJ dysfunction" OR "TMJ disease" OR " TMJ syndrome")) | | 36,803 |
| 2. Systematic review | |  |
| (TITLE-ABS-KEY ("systematic review" OR "systematic literature review" OR "systematic scoping review" OR "systematic narrative review" OR "systematic qualitative review" OR "systematic evidence review" OR "systematic quantitative review" OR "systematic meta-review" OR "systematic critical review" OR "systematic mixed studies review" OR "systematic mapping review" OR "cochrane review" OR "systematic search and review" OR "systematic integrative review")) | | 538,872 |
| 1 AND 2 | | 877 |

| LILACS via VHL |  | |  | | |
| --- | --- | --- | --- | --- | --- |
| 1. TMJD | | |  | | |
| (mh:("Craniomandibular Disorders" OR "Temporomandibular Joint Disorders" OR "Temporomandibular Joint Dysfunction Syndrome") OR (tmd OR tmjd OR tmj disorder OR tmj dysfunction OR tmj disease OR tmj syndrome OR temporomandibular joint disorder OR temporomandibular disorder OR temporomandibular joint disease OR temporomandibular joint dysfunction syndrome OR temporomandibular joint syndrome OR temporomandibular joint dysfunction OR temporomandibular dysfunction OR craniomandibular disorder OR craniomandibular dysfunction)) | | | | 2 426 | |
| 2. Systematic review | |  | | | |
| (ti:("systematic review" OR "systematic literature review" OR "systematic scoping review" OR "systematic narrative review" OR "systematic qualitative review" OR "systematic evidence review" OR "systematic quantitative review" OR "systematic meta-review" OR "systematic critical review" OR "systematic mixed studies review" OR "systematic mapping review" OR "cochrane review" OR "systematic search and review" OR "systematic integrative review")) OR (pt: review OR "systematic review") | | 1 069 374 | | | |
| 1 AND 2 | | | | | 261 |
| 1 AND type_of_study:("systematic_review" OR "evaluation_study") | | | | | 132 |
| 3 OR 4 | | | | | 332 |

| Cochrane via Wiley |  |  |
| --- | --- | --- |
| 1. MeSH descriptor: [Craniomandibular Disorders] explode all trees | | 1 079 |
| 2. TMJD | |  |
| (TMD OR TMJD OR TMJ disorder OR TMJ dysfunction OR TMJ disease OR TMJ syndrome OR TMJ disorders OR TMJ diseases OR TMJ syndromes OR TMJ dysfunctions OR temporomandibular joint disorders OR temporomandibular joint disorder OR temporomandibular disorders OR temporomandibular disorder OR temporomandibular joint disease OR temporomandibular joint diseases OR temporomandibular joint dysfunction syndrome OR temporomandibular joint syndrome OR temporomandibular joint dysfunction syndromes OR temporomandibular joint syndromes OR temporomandibular joint dysfunction OR temporomandibular joint dysfunctions OR temporomandibular dysfunction OR temporomandibular dysfunctions OR Craniomandibular Disorders OR Craniomandibular Disorder OR Craniomandibular Dysfunction OR Craniomandibular Dysfunctions) OR ((facial OR jaw OR orofacial OR craniofacial OR trigem*) AND (pain)):ti,ab,kw | | 7 091 |
| 1 OR 2 | | 7 091 |
| Cochrane Database of Systematic Reviews | | 104 |

**Appendix 2.** Reference list of studies for which the full text was not found (n=7) and studies excluded during full-text assessment (n=378)

1. Abdel Shaheed C, Maher CG, et al (2019). Efficacy and safety of low-dose codeine-containing combination analgesics for pain: Systematic review and meta-analysis Clinical Journal of Pain 35:836-43.
2. Abdul AM, Gilligan EM, et al (2023). Effectiveness of self-management interventions for chronic orofacial pain: A systematic review and meta-analysis. Journal of Oral Rehabilitation 50:1124-35.
3. Abdul NS, Minervini G (2023). Prevalence of Temporomandibular Disorders in Orthognathic Surgery patients: A systematic review conducted according to PRISMA guidelines and the Cochrane Handbook for Systematic Reviews of Interventions J Oral Rehabil 50:1093-100.
4. Aggarwal VR, Fu Y, et al (2019). Psychosocial interventions for the management of chronic orofacial pain. Cochrane Database of Systematic Reviews 11:CD011195.
5. Agurto P J, Mardones M M, et al (2004). Hiperplasia coronoídea Rev. otorrinolaringol. cir. cabeza cuello 64:32-8.
6. Ahmad SA, Hasan S, et al (2021). Low-level laser therapy in temporomandibular joint disorders: a systematic review J Med Life 14:148-64.
7. Ahmadi DP (2020). Evaluation of the effective orthognathic surgery on pre-existing temporomandibular disorders (Tmds) in patients with malocclusion: Systematic review meta-analysis International Journal of Pharmaceutical Research 12:1839-43.
8. Akinbami BO (2011). Evaluation of the mechanism and principles of management of temporomandibular joint dislocation. Systematic review of literature and a proposed new classification of temporomandibular joint dislocation Head Face Med 7:10.
9. Al-Ani MZ, Davies SJ, et al (2016). WITHDRAWN: Stabilisation splint therapy for temporomandibular pain dysfunction syndrome Cochrane Database Syst Rev Cd002778.
10. Al-Baghdadi M, Durham J, et al (2014). Timing interventions in relation to temporomandibular joint closed lock duration: a systematic review of 'locking duration' J Oral Rehabil 41:24-58.
11. Al-Baghdadi M, Durham J, et al (2014). TMJ Disc Displacement without Reduction Management: A Systematic Review J Dent Res 93:37s-51s.
12. Al-Hamed FS, Hijazi A, et al (2021). Platelet Concentrate Treatments for Temporomandibular Disorders: A Systematic Review and Meta-analysis JDR Clin Trans Res 6:174-83.
13. Al-Moraissi EA (2015a). Arthroscopy versus arthrocentesis in the management of internal derangement of the temporomandibular joint: a systematic review and meta-analysis Int J Oral Maxillofac Surg 44:104-12.
14. Al-Moraissi EA (2015b). Open versus arthroscopic surgery for the management of internal derangement of the temporomandibular joint: A meta-analysis of the literature International Journal of Oral and Maxillofacial Surgery 44:763-70.
15. Al-Moraissi EA, Conti PCR, et al (2022). Correction to: The hierarchy of different treatments for myogenous temporomandibular disorders: a systematic review and network meta‑analysis of randomized clinical trials Oral Maxillofac Surg 26:679.
16. Al-Moraissi EA, Conti PCR, et al (2022). The hierarchy of different treatments for myogenous temporomandibular disorders: a systematic review and network meta-analysis of randomized clinical trials Oral Maxillofac Surg 26:519-33.
17. Al-Moraissi EA, Wolford LM, et al (2017). Does Orthognathic Surgery Cause or Cure Temporomandibular Disorders? A Systematic Review and Meta-Analysis J Oral Maxillofac Surg 75:1835-47.
18. Al-Riyami S, Cunningham SJ, et al (2009). Orthognathic treatment and temporomandibular disorders: a systematic review. Part 2. Signs and symptoms and meta-analyses Am J Orthod Dentofacial Orthop 136:626.e1-16, discussion
19. Alajbeg I, Živković K, et al (2015). [THE ROLE OF STABILIZATION SPLINT IN THE TREATMENT OF TEMPOROMANDIBULAR DISORDERS] Acta Med Croatica 69:33-43.
20. Alam MK, Abutayyem H, et al (2023). The Impact of Temporomandibular Disorders on Orthodontic Management: A Systematic Review and Meta-Analysis Cureus 15:e44243.
21. Albagieh H, Alomran I, et al (2023). Occlusal splints-types and effectiveness in temporomandibular disorder management Saudi Dental Journal 35:70-9.
22. Algabri RS, Alqutaibi AY (2017). No Evidence Suggests that the Clinical Effectiveness of Conventional Occlusal Splints is Superior to That of Psychosocial Interventions for Myofascial Tempromandibular Disorders Pain Journal of Evidence-Based Dental Practice 17:399-401.
23. Almășan O, Hedeșiu M, et al (2023). Psoriatic arthritis of the temporomandibular joint: A systematic review J Oral Rehabil 50:243-55.
24. Alsarhan J, El Feghali R, et al (2022). Can Photobiomodulation Support the Management of Temporomandibular Joint Pain? Molecular Mechanisms and a Systematic Review of Human Clinical Trials Photonics 9.
25. Alves BM, Macedo CR, et al (2013). Mandibular manipulation for the treatment of temporomandibular disorder J Craniofac Surg 24:488-93.
26. Alves RdLBR, Silva PFdS, et al (2010). A eficácia dos recursos fisioterapêuticos no ganho da amplitude de abertura bucal em pacientes com disfunções craniomandibulares Rev. odontol. UNESP (Online) 39:55-61.
27. Amat P, Tran Lu YE (2023). [Orofacial myofunctional reeducation assisted by a prefabricated reeducation appliance: a systematic review of the literature] Orthod Fr 94:131-61.
28. Ansari S, Charantimath S, et al (2022). Comparative efficacy of low-level laser therapy (LLLT) to TENS and therapeutic ultrasound in management of TMDs: a systematic review & meta-analysis Cranio 1-10.
29. Araneda P, Oyarzo JF, et al (2013). Intervención psicológica en trastornos temporomandibulares: revision narrativa J. oral res. (Impresa) 2:86-90.
30. Arbildo-Vega H, Alay-Baca V, et al (2021). Efectividad de los concentrados plaquetarios en el tratamiento de los desórdenes temporomandibulares Rev. cuba. estomatol 58:e3053-e.
31. Argueta-Figueroa L, Flores-Mejía LA, et al (2022). Nonpharmacological Interventions for Pain in Patients with Temporomandibular Joint Disorders: A Systematic Review Eur J Dent 16:500-13.
32. Armijo-Olivo S, Michelotti A, et al (2015). Advances in physical therapy interventions for managing orofacial pain.
33. Armijo-Olivo S, Pitance L, et al (2016). Effectiveness of Manual Therapy and Therapeutic Exercise for Temporomandibular Disorders: Systematic Review and Meta-Analysis Phys Ther 96:9-25.
34. Arribas-Pascual M, Hernández-Hernández S, et al (2023). Effects of Physiotherapy on Pain and Mouth Opening in Temporomandibular Disorders: An Umbrella and Mapping Systematic Review with Meta-Meta-Analysis J Clin Med 12.
35. Askar H, Aronovich S, et al (2021). Is Arthroscopic Disk Repositioning Equally Efficacious to Open Disk Repositioning? A Systematic Review Journal of Oral and Maxillofacial Surgery 79:2030-41.e2.
36. Asquini G, Pitance L, et al (2022). Effectiveness of manual therapy applied to craniomandibular structures in temporomandibular disorders: A systematic review J Oral Rehabil 49:442-55.
37. Asquini G, Rushton A, et al (2021). The effectiveness of manual therapy applied to craniomandibular structures in the treatment of temporomandibular disorders: protocol for a systematic review. Syst Rev. 10(1):70.
38. Assis TdO, Soares MdS, et al (2012). O uso do laser na reabilitação das desordens temperomandibulares Fisioter. Mov 25:453-9.
39. Ateneo Argentino de O (2007). Resumen de revisiones sistemáticas basadas en la evidencia de los desórdenes temporomandibulares Rev. Ateneo Argent. Odontol 46:44-50.
40. Ávila-Curiel BX, Gómez-Aguirre JN, et al (2020). Complementary interventions for pain in patients with temporomandibular joint disorders: a systematic review Revista Internacional de Acupuntura 14:151-9.
41. Awan KH, Patil S, et al (2019). Botulinum toxin in the management of myofascial pain associated with temporomandibular dysfunction J Oral Pathol Med 48:192-200.
42. Azzopardi EA, Whitaker IS (2010). Acute pain management during facial injection of botulinum toxin: an evidence-based systematic review Plast Reconstr Surg 125:162e-4e.
43. Bach E, Sigaux N, et al (2022). Reasons for failure of total temporomandibular joint replacement: a systematic review and meta-analysis Int J Oral Maxillofac Surg 51:1059-68.
44. Bahgat MM, Abdel-Hamid AM (2023). Is dextrose prolotherapy beneficial in the management of temporomandibular joint internal derangement? A systematic review Cranio 1-9.
45. Bankersen CN, Costa CdC, et al (2021). Terapia fonoaudiológica nas disfunÒ«ões temporomandibulares (DTM): uma revisão de literatura Distúrb. comun 33:239-48.
46. Barbosa MCSA, Kinoshita ÂMO, et al (2018). Biomateriais utilizados na artroplastia parcial ou total da articulação temporomandibular: uma revisão de literatura Rev. Salusvita (Online) 37:389-403.
47. Barry F, Chai F, et al (2021). Systematic review of studies on drug-delivery systems for management of temporomandibular-joint osteoarthritis J Stomatol Oral Maxillofac Surg.
48. Barry F, Chai F, et al (2022). Systematic review of studies on drug-delivery systems for management of temporomandibular-joint osteoarthritis J Stomatol Oral Maxillofac Surg 123:e336-e41.
49. Batista JFOL, Moraes SLD, et al (2022a). Are exercises with or without occlusal splints more effective in the reduction of pain in patients with temporomandibular disorders of myogenic origin? A systematic review Journal of Applied Oral Science 30.
50. Batista RR, Farias CVdS, et al (2022b). Eficácia do tratamento fisioterapêutico em mulheres com disfunções temporomandibulares: uma revisão integrativa da literatura Fisioter. Bras 23:173-87.
51. Bavarian R, Khawaja SN, et al (2021). The efficacy of microcurrent electrical nerve stimulation in treating masticatory myofascial pain: A systematic review and meta-analysis Cranio 1-7.
52. Beecroft EV, Penlington C, et al (2021). Pharmacological interventions for painful persistent temporomandibular disorders (TMD). Cochrane Database of Systematic Reviews 2021, Issue 12.
53. Bermell-Baviera A, Bellot-Arcís C, et al (2016). Effects of mandibular advancement surgery on the temporomandibular joint and muscular and articular adaptive changes--a systematic review Int J Oral Maxillofac Surg 45:545-52.
54. Bessa-Nogueira RV, Vasconcelos BCE, et al (2008). The methodological quality of systematic reviews comparing temporomandibular joint disorder surgical and non-surgical treatment BMC Oral Health 8.
55. Beyer L, Vinzelberg S, et al (2022). Evidence (-based medicine) in manual medicine/manual therapy—a summary review Manuelle Medizin 60:203-23.
56. Bou CM, Sarrión ÓG (2022). Arthrocentesis of the temporomandibular joint and corticosteroids. A systematic review Revista Espanola de Cirugia Oral y Maxilofacial 44:30-9.
57. Bouchard C, Goulet JP, et al (2017). Temporomandibular Lavage Versus Nonsurgical Treatments for Temporomandibular Disorders: A Systematic Review and Meta-Analysis J Oral Maxillofac Surg 75:1352-62.
58. Bousnaki M, Bakopoulou A, et al (2018). Platelet-rich plasma for the therapeutic management of temporomandibular joint disorders: a systematic review Int J Oral Maxillofac Surg 47:188-98.
59. Brantingham JW, Cassa TK, et al (2013). Manipulative and multimodal therapy for upper extremity and temporomandibular disorders: a systematic review J Manipulative Physiol Ther 36:143-201.
60. Brighenti N, Battaglino A, et al (2023). Effects of an Interdisciplinary Approach in the Management of Temporomandibular Disorders: A Scoping Review Int J Environ Res Public Health 20.
61. Brignardello-Petersen R (2019). Important uncertainty regarding the effects of botulinum toxin in patients with temporomandibular disorders or bruxism owing to serious limitations of a systematic review Journal of the American Dental Association 150:e175.
62. Brochado FT, Jesus LHd, et al (2019). Non-invasive therapies for management of temporomandibular disorders: a systematic review Clin. biomed. res 39:230-43.
63. Butts R, Dunning J, et al (2017). Conservative management of temporomandibular dysfunction: A literature review with implications for clinical practice guidelines (Narrative review part 2) Journal of Bodywork and Movement Therapies 21:541-8.
64. Calatrava Oramas LA (2014). Mínima invasión en la terapia de disfunciones oclusales Acta odontol. venez 52.
65. Calderon PdS (2008). Avaliação da eficácia de um programa remediativo cognitivo-comportamental e do uso de amitriptilina em pacientes portadores de disfunções temporomandibulares crônicas, pp. 131-.
66. Calixtre LB, Moreira RF, et al (2015). Manual therapy for the management of pain and limited range of motion in subjects with signs and symptoms of temporomandibular disorder: a systematic review of randomised controlled trials J Oral Rehabil 42:847-61.
67. Cascos-Romero J, Vázquez-Delgado E, et al (2009). The use of tricyclic antidepressants in the treatment of temporomandibular joint disorders: systematic review of the literature of the last 20 years Med Oral Patol Oral Cir Bucal 14:E3-7.
68. Castaño-Joaqui OG, Muñoz-Guerra MF, et al (2017). Current status of viscosupplementation with hyaluronic acid in the treatment of temporomandibular disorders: A systematic review Revista Espanola de Cirugia Oral y Maxilofacial 39:213-20.
69. Chalco Valdivia AV, López Flores AI (2019). Consideraciones actuales sobre el uso de férulas oclusales en rehabilitación oral: una revisión crítica Rev. cient. odontol 7:157-67.
70. Chang WD, Lee CL, et al (2014). A meta-analysis of clinical effects of low-level laser therapy on temporomandibular joint pain Journal of Physical Therapy Science 26:1297-300.
71. Chauvel-Lebret D, Leroux A, et al (2013). [Relations between orthognathic surgery and temporomandibular disorders: a systematic review] Orthod Fr 84:169-83.
72. Chen J, Li Y, et al (2021). Tuina therapy for temporomandibular joint disorder syndrome: A protocol for systematic review and meta-analysis. Medicine (Baltimore). 100:e24202.
73. Chęciński M, Chęcińska K, et al (2022a). Treatment of Mandibular Hypomobility by Injections into the Temporomandibular Joints: A Systematic Review of the Substances Used J Clin Med 11.
74. Chęciński M, Chęcińska K, et al (2022b). Autologous Stem Cells Transplants in the Treatment of Temporomandibular Joints Disorders: A Systematic Review and Meta-Analysis of Clinical Trials Cells 11.
75. Chęciński M, Chęcińska K, et al (2023). Autologous Blood Injections in Temporomandibular Hypermobility: A Systematic Review J Clin Med 12.
76. Chęciński M, Chęcińska K, et al (2023). Intra-Articular Injections into the Inferior versus Superior Compartment of the Temporomandibular Joint: A Systematic Review and Meta-Analysis J Clin Med 12.
77. Chen YW, Chiu YW, et al (2015). Botulinum toxin therapy for temporomandibular joint disorders: a systematic review of randomized controlled trials Int J Oral Maxillofac Surg 44:1018-26.
78. Cho SH, Whang WW (2010). Acupuncture for temporomandibular disorders: a systematic review J Orofac Pain 24:152-62.
79. Christidis N, Lindström Ndanshau E, et al (2019). Prevalence and treatment strategies regarding temporomandibular disorders in children and adolescents-A systematic review J Oral Rehabil 46:291-301.
80. Coronel-Zubiate FT, Marroquín-Soto C, et al (2022). Association between orthodontic treatment and the occurrence of temporomandibular disorders: A systematic review and meta-analysis J Clin Exp Dent 14:e1032-e43.
81. Craane B, De Laat A, et al (2018). Physical therapy for the management of patients with temporomandibular disorders and related pain Cochrane Database of Systematic Reviews 2018.
82. da Silva FM, Serrano RV, et al (2023a). Impact of different anesthetic approaches on the outcomes of temporomandibular joint arthrocentesis: a systematic review Oral Maxillofac Surg 27:591-600.
83. da Silva MT, Silva C, et al (2023b). Effectiveness of Physical Therapy Interventions for Temporomandibular Disorders Associated with Tinnitus: A Systematic Review J Clin Med 12.
84. da Silveira RB, Ferreira I, et al (2022). Effect of photobiomodulation treatment on pain control in patients with temporomandibular dysfunction disorder: systematic review Cranio 1-11.
85. Dall, Antonia M, et al (2013). Dor miofascial dos músculos da mastigação e toxina botulínica Rev. dor 14:52-7.
86. Dall, Magro AK, et al (2015). Aplicações da toxina botulínica em odontologia Rev. Salusvita (Online) 34.
87. Dammling C, Abramowicz S, et al (2022). The use of pharmacologic agents in the management of temporomandibular joint disorder Frontiers of Oral and Maxillofacial Medicine 4.
88. Davoudi A, Khaki H, et al (2018). Is arthrocentesis of temporomandibular joint with corticosteroids beneficial? A systematic review Med Oral Patol Oral Cir Bucal 23:e367-e75.
89. de Almeida VL, Vitorino Nde S, et al (2016). Stability of treatments for recurrent temporomandibular joint luxation: a systematic review Int J Oral Maxillofac Surg 45:304-7.
90. de Castro-Carletti EM, Müggenborg F, et al (2023). Effectiveness of electrotherapy for the treatment of orofacial pain: A systematic review and meta-analysis Clin Rehabil 37:891-926.
91. de Freitas RF, Mezzomo LA, et al (2013). Efficacy of relaxation techniques in Treatment of Temporomandibular Disorders: A systematic review. Revista Dor 14:211-15.
92. de la Barra Ortiz HA, Cofré CJ, et al (2023). Efficacy of diadynamic currents in the treatment of musculoskeletal pain: a systematic review Physiotherapy Quarterly 31:1-19.
93. De la Torre Canales G, Poluha RL, et al (2019). Botulinum toxin type A applications for masticatory myofascial pain and trigeminal neuralgia: what is the evidence regarding adverse effects? Clin Oral Investig 23:3411-21.
94. de Melo LA, Bezerra de Medeiros AK, et al (2020). Manual Therapy in the Treatment of Myofascial Pain Related to Temporomandibular Disorders: A Systematic Review J Oral Facial Pain Headache 34:141-8.
95. De Meurechy N, Mommaerts MY (2018). Alloplastic temporomandibular joint replacement systems: a systematic review of their history Int J Oral Maxillofac Surg 47:743-54.
96. de Oliveira-Souza AIS, Mohamad N, et al (2023). What are the best parameters of low-level laser therapy to reduce pain intensity and improve mandibular function in orofacial pain? A systematic review and meta-analysis Disabil Rehabil 45:3219-37.
97. de Souza RF, Lovato da Silva CH, et al (2012). Interventions for the management of temporomandibular joint osteoarthritis Cochrane Database Syst Rev 2012:Cd007261.
98. Del Vecchio A, Porrini M, et al (2023). Photobiomodulation in odontostomatology Dental Cadmos 91:2-22.
99. Derwich M, Górski B, et al (2023). Oral Glucosamine in the Treatment of Temporomandibular Joint Osteoarthritis: A Systematic Review Int J Mol Sci 24.
100. Derwich M, Mitus-Kenig M, et al (2021). Mechanisms of Action and Efficacy of Hyaluronic Acid, Corticosteroids and Platelet-Rich Plasma in the Treatment of Temporomandibular Joint Osteoarthritis-A Systematic Review Int J Mol Sci 22.
101. Desai H, Pande N, et al (2022). Comparison of surgical outcomes related to interpositional arthroplasty materials used in patients with temporomandibular joint ankylosis: a systematic review and meta-analysis Br J Oral Maxillofac Surg 60:1023-34.
102. Di Francesco F, Lanza A, et al (2022). Application of Botulinum Toxin in Temporomandibular Disorders: A Systematic Review of Randomized Controlled Trials (RCTs) Applied Sciences (Switzerland) 12.
103. Dickerson SM, Weaver JM, et al (2017). The effectiveness of exercise therapy for temporomandibular dysfunction: a systematic review and meta-analysis Clin Rehabil 31:1039-48.
104. Ding L, Chen R, et al (2022). The effect of functional mandibular advancement for adolescent patients with skeletal class II malocclusion on the TMJ: a systematic review and meta-analysis BMC Oral Health 22:51.
105. Dinsdale A, Costin B, et al (2022). What conservative interventions improve bite function in those with temporomandibular disorders? A systematic review using self-reported and physical measures J Oral Rehabil 49:456-75.
106. Dommerholt J, Chou LW, et al (2017). A critical overview of the current myofascial pain literature – June 2017 Journal of Bodywork and Movement Therapies 21:673-83.
107. Dommerholt J, Finnegan M, et al (2016). A critical overview of the current myofascial pain literature – January 2016 Journal of Bodywork and Movement Therapies 20:156-67.
108. Dziedzic K, Jordan JL, et al (2008). Land- and water-based exercise therapies for musculoskeletal conditions Best Practice and Research: Clinical Rheumatology 22:407-18.
109. El-Kahky AM, Hamdy TAH, et al (2022). Meta Analytical Study of the Role of Intramuscular Botulinum A Toxin Injection in the Treatment of Temporomandibular Joint (TMJ) Disorders Egyptian Journal of Ear, Nose, Throat and Allied Sciences 23.
110. Elbarbary M, Sgro A, et al (2022). The applications of ultrasound, and ultrasonography in dentistry: a scoping review of the literature Clinical oral investigations 26:2299-316.
111. Emérito TM, Silva JAS, et al (2022). O uso da bandagem elástica adesiva para alívio da dor no tratamento das disfunções temporomandibulares: revisão sistemática com metanálise Audiol., Commun. res 27:e2631-e.
112. Ernst E, White AR (1999). Acupuncture as a treatment for temporomandibular joint dysfunction: a systematic review of randomized trials Arch Otolaryngol Head Neck Surg 125:269-72.
113. Fakhry H, Abd-Elwahab Radi I (2018). Limited Evidence Suggests No Benefit of Temporomandibular Joint Lavage Over Conservative Treatment for Temporomandibular Joint Pain and Dysfunction J Evid Based Dent Pract 18:157-8.
114. Farook TH, Dudley J (2023). Neuroendocrine Influencers and Associated Factors That Shape Jaw Movement and Growth in Temporomandibular Joint Disorder Management: A Systematic Review of Clinical and Radiographic Evidence J Pers Med 13.
115. Farshidfar N, Farzinnia G, et al (2023). The Effect of Photobiomodulation on Temporomandibular Pain and Functions in Patients With Temporomandibular Disorders: An Updated Systematic Review of the Current Randomized Controlled Trials J Lasers Med Sci 14:e24.
116. Fassina MT, Jóias RM, et al (2016). Toxina botulínica tipo A nas DTM musculares: há eficácia? Odonto (Säo Bernardo do Campo) 24:1-13.
117. Feng J, Luo M, et al (2019). The treatment modalities of masticatory muscle pain a network meta-analysis Medicine 98:e17934.
118. Fernandes AC, Duarte Moura DM, et al (2017). Acupuncture in Temporomandibular Disorder Myofascial Pain Treatment: A Systematic Review J Oral Facial Pain Headache 31:225-32.
119. Fernandes TdF (2015). Acupuntura na analgesia da disfunção temporomandibular: revisão integrativa da literatura, pp. 59-.
120. Fernández-Hernández S, Brenes-Ortega L, et al (2017). Inyecciones Intraarticulares de Ácido Hialurónico como Alternativa a los Corticoesteroides enel Tratamiento de la Osteoartritis de la Articulación Témporomandibular: Estudio de Revisión Sistemática Int. j. odontostomatol. (Print) 11:157-64.
121. Fernandez-Vial D, Silva-Arce F, et al (2021). Effectiveness of hyaluronic acid in the treatment of degenerative diseases of the temporomandibular joint: literature review J. oral res. (Impresa) 10:1-10.
122. Ferreira N, Masterson D, et al (2018). Efficacy of viscosupplementation with hyaluronic acid in temporomandibular disorders: A systematic review J Craniomaxillofac Surg 46:1943-52.
123. Ferreira NR, Junqueira YN, et al (2019). The efficacy of transcranial direct current stimulation and transcranial magnetic stimulation for chronic orofacial pain: A systematic review PLoS One 14:e0221110.
124. Ferreira SLdS, Cunha DAd, et al (2021). The use of photobiomodulation for the muscles of head and neck: an integrative review Audiol., Commun. res 26:e2552-e.
125. Ferrillo M, Ammendolia A, et al (2022a). Efficacy of rehabilitation on reducing pain in muscle-related temporomandibular disorders: A systematic review and meta-analysis of randomized controlled trials J Back Musculoskelet Rehabil 35:921-36.
126. Ferrillo M, Marotta N, et al (2022). Effects of Occlusal Splints on Spinal Posture in Patients with Temporomandibular Disorders: A Systematic Review Healthcare (Basel) 10.
127. Ferrillo M, Nucci L, et al (2022b). Efficacy of conservative approaches on pain relief in patients with temporomandibular joint disorders: a systematic review with network meta-analysis Cranio 1-17.
128. Fertout A, Manière-Ezvan A, et al (2022). Management of temporomandibular disorders with transcutaneous electrical nerve stimulation: A systematic review Cranio 40:217-28.
129. Fisher E, Law E, et al (2018). Psychological therapies (remotely delivered) for the management of chronic and recurrent pain in children and adolescents. Cochrane Database of Systematic Reviews 4:CD011118.
130. Florjanski W, Malysa A, et al (2019). Evaluation of Biofeedback Usefulness in Masticatory Muscle Activity Management-A Systematic Review J Clin Med 8.
131. Forssell H, Kalso E (2004). Application of principles of evidence-based medicine to occlusal treatment for temporomandibular disorders: are there lessons to be learned? J Orofac Pain 18:9-22; discussion 3.
132. Fricton J (2006). Current evidence providing clarity in management of temporomandibular disorders: summary of a systematic review of randomized clinical trials for intra-oral appliances and occlusal therapies J Evid Based Dent Pract 6:48-52.
133. Furlan AD (2011). Evidence for interferential current to treat musculoskeletal pain remains weak Clinical Journal of Sport Medicine 21:278-9.
134. Furlan RMMM (2015). O uso da crioterapia no tratamento das disfunções temporomandibulares Rev. CEFAC 17:648-55.
135. Furlan RMMM, Giovanardi RS, et al (2015). The use of superficial heat for treatment of temporomandibular disorders: an integrative review CoDAS 27:207-12.
136. Gadhia K, Walmsley D (2009). The therapeutic use of botulinum toxin in cervical and maxillofacial conditions Evidence-Based Dentistry 10:53.
137. Gallardo Leyva CA, Ascanio Balderas M, et al (2018). Guarda oclusal gnatológica: técnica de elaboración, procesado y polimerizado por microondas Rev. ADM 75:228-36.
138. Gallego Duque D, Hincapie Mora C (1988). Sindrome de Disfuncion Meniscal Intra-articular de la Articulacion Temporomandibular: con el Complemento de un Caso Clinico, pp. XVI,222-XVI,.
139. Ganem A, Rossouw PE, et al (2023). Antinociceptive Efficacy of Shamanic Healing for the Management of Temporomandibular Disorders: An Evidence-Based Review Journal of Religion and Health.
140. Goiato MC, da Silva EV, et al (2016). Are intra-articular injections of hyaluronic acid effective for the treatment of temporomandibular disorders? A systematic review Int J Oral Maxillofac Surg 45:1531-7.
141. Goker F, Russillo A, et al (2021). Evaluation of Arthrocentesis with hyaluronic acid injections for management of temporomandibular disorders: a systematic review and case series J Biol Regul Homeost Agents 35:21-35.
142. Goldberg P, Gutiérrez AM, et al (2003). Conceptos actuales del tratamiento de las alteraciones en la articulación temporomandibular Rev. ADM 60:225-8.
143. Gonzalez LV, López JP, et al (2023). Diagnosis and management of temporomandibular joint synovial chondromatosis: A systematic review Journal of Cranio-Maxillofacial Surgery 51:551-9.
144. González Mendoza E, Hernández Calva A (2007). Anquilosis temporomandibular. Revisión de la literatura: protocolo de manejo e informe de un caso Rev. ADM 64:238-43.5
145. González-Sánchez B, García Monterey P, et al (2023). Temporomandibular Joint Dysfunctions: A Systematic Review of Treatment Approaches J Clin Med 12.
146. Gopi I, Maragathavalli G, et al (2021). Efficacy of transcutaneous electric nerve stimulation over systemic pharmacotherapy in the management of temporomandibular joint disorders – A systematic review and meta-analysis Journal of Indian Academy of Oral Medicine and Radiology 33:321-7.
147. Grossman S, Tan H, et al (2022). Cannabis and orofacial pain: a systematic review Br J Oral Maxillofac Surg 60:e677-e90.
148. Grossmann E (2012). Técnicas de artrocentese aplicadas às disfunções artrogênicas da articulação temporomandibular Rev. dor 13:374-81.
149. Grossmann E, Grossmann TK (2011). Cirurgia da articulação temporomandibular Rev. dor 12.
150. Grossmann E, Tambara JS, et al (2012). O uso da estimulação elétrica nervosa transcutânea na disfunção temporomandibular Rev. dor 13:271-6.
151. Guarda-Nardini L, De Almeida AM, et al (2021). Arthrocentesis of the Temporomandibular Joint: Systematic Review and Clinical Implications of Research Findings J Oral Facial Pain Headache 35:17-29.
152. Guarda-Nardini L, Trojan D, et al (2017). Management of temporomandibular joint degenerative disorders with human amniotic membrane: Hypothesis of action Medical Hypotheses 104:68-71.
153. Guo C, Shi Z, et al (2015). WITHDRAWN: Arthrocentesis and lavage for treating temporomandibular joint disorders Cochrane Database Syst Rev Cd004973.
154. Gutiérrez IQ, Sábado-Bundó H, et al (2022). Intraarticular injections of platelet rich plasma and plasma rich in growth factors with arthrocenthesis or arthroscopy in the treatment of temporomandibular joint disorders: A systematic review J Stomatol Oral Maxillofac Surg 123:e327-e35.
155. Haddad C, Zoghbi A, et al (2023). Platelet-rich plasma injections for the treatment of temporomandibular joint disorders: A systematic review J Oral Rehabil 50:1330-9.
156. Häggman-Henrikson B, Alstergren P, et al (2017). Pharmacological treatment of oro-facial pain - health technology assessment including a systematic review with network meta-analysis J Oral Rehabil 44:800-26.
157. Haigler MC, Abdulrehman E, et al (2018). Use of platelet-rich plasma, platelet-rich growth factor with arthrocentesis or arthroscopy to treat temporomandibular joint osteoarthritis: Systematic review with meta-analyses J Am Dent Assoc149:940-52.e2.
158. Hanna R, Dalvi S, et al (2021). Role of Photobiomodulation Therapy in Modulating Oxidative Stress in Temporomandibular Disorders. A Systematic Review and Meta-Analysis of Human Randomised Controlled Trials Antioxidants (Basel) 10.
159. Harrison L, Jones NS (2013). Intranasal contact points as a cause of facial pain or headache: a systematic review Clin Otolaryngol 38:8-22.
160. Herpich CM, Amaral AP, et al (2015). Analysis of laser therapy and assessment methods in the rehabilitation of temporomandibular disorder: a systematic review of the literature J Phys Ther Sci 27:295-301.
161. Herranz-Aparicio J, Vázquez-Delgado E, et al (2013). The use of low level laser therapy in the treatment of temporomandibular joint disorders. Review of the literature Medicina Oral, Patologia Oral y Cirugia Bucal 18:e603-e12.
162. Herrera-Valencia A, Ruiz-Muñoz M, et al (2020). Effcacy of Manual Therapy in TemporomandibularJoint Disorders and Its Medium-and Long-TermEffects on Pain and Maximum Mouth Opening: A Systematic Review and Meta-Analysis J Clin Med 9.
163. Herrero Babiloni A, Guay S, et al (2018). Non-invasive brain stimulation in chronic orofacial pain: a systematic review J Pain Res 11:1445-57.
164. Hsing LC, Ilankovan V (2020). RE: Botulinum toxin in the management of temporomandibular disorders: a systematic review British Journal of Oral and Maxillofacial Surgery 58:1213-4.
165. Hu Y, Liu S, et al (2023a). Arthrocentesis vs conservative therapy for the management of TMJ disorders: A systematic review and meta-analysis J Stomatol Oral Maxillofac Surg 124:101283.
166. Hu Y, Zhang X, et al (2023b). Ultrasound-guided vs conventional arthrocentesis for management of temporomandibular joint disorders: A systematic review and meta-analysis Cranio 41:264-73.
167. Huggins T, Boras AL, et al (2012). Clinical effectiveness of the activator adjusting instrument in the management of musculoskeletal disorders: a systematic review of the literature J Can Chiropr Assoc 56:49-57.
168. Idáñez-Robles AM, Obrero-Gaitán E, et al (2023). Exercise therapy improves pain and mouth opening in temporomandibular disorders: A systematic review with meta-analysis Clin Rehabil 37:443-61.
169. Iturriaga V, Bornhardt T, et al (2017a). Effect of hyaluronic acid on the regulation of inflammatory mediators in osteoarthritis of the temporomandibular joint: a systematic review Int J Oral Maxillofac Surg 46:590-5.
170. Iturriaga V, Vásquez B, et al (2017b). Role of hyaluronic acid in the homeostasis and therapeutics of temporomandibular joint osteoarthritis Int. j. morphol 35:870-6.
171. Ivorra-Carbonell L, Montiel-Company JM, et al (2016). Impact of functional mandibular advancement appliances on the temporomandibular joint - a systematic review Med Oral Patol Oral Cir Bucal 21:e565-72.
172. Jamali Z, Hadilou N, et al (2022). Effect of the posterior stop on temporomandibular disorders: A systematic review J Dent Res Dent Clin Dent Prospects 16:147-52.
173. Januzzi E, Nasri-Heir C, et al (2013). Combined palliative and anti-inflammatory medications as treatment of temporomandibular joint disc displacement without reduction: a systematic review Cranio 31:211-25.
174. Jara Armijos JI, Hidalgo Andrade B, et al (2020). Effectiveness of hialuronic acid in temporomandibular disorders treatment. A systematic review Avances en Odontoestomatologia 36:35-47.
175. Javed F, Bello-Correa FO, et al (2021). Anti-nociceptive efficacy of essential oil-based extracts for the management of orofacial pain: a systematic review of available evidence Eur Rev Med Pharmacol Sci 25:7323-32.
176. Jazayeri HE, Lopez J, et al (2023). Comparative Benefits of Open versus Closed Reduction of Condylar Fractures: A Systematic Review and Meta-Analysis Plast Reconstr Surg 151:664e-72e.
177. Jedel E, Carlsson J (2003). Biofeedback, Acupuncture and Transcutaneous Electric Nerve Stimulation in the Management of Temperomandibular Disorders: A Systematic Review Physical Therapy Reviews 8:217-23.
178. Jing G, Zhao Y, et al (2021). Effects of different energy density low-level laser therapies for temporomandibular joint disorders patients: a systematic review and network meta-analysis of parallel randomized controlled trials Lasers Med Sci 36:1101-8.
179. Jung A, Shin BC, et al (2011). Acupuncture for treating temporomandibular joint disorders: a systematic review and meta-analysis of randomized, sham-controlled trials J Dent 39:341-50.
180. Kamińska A, Dalewski B, et al (2020). The Usefulness of the Pressure Algometer in the Diagnosis and Treatment of Orofacial Pain Patients: A Systematic Review Occup Ther Int 2020:5168457.
181. Katsnelson A, Markiewicz MR, et al (2012). Operative management of temporomandibular joint ankylosis: a systematic review and meta-analysis. J Oral Maxillofac Surg. 70:531-6.
182. Kietrys DM, Palombaro KM, et al (2014). Dry needling for management of pain in the upper quarter and craniofacial region Curr Pain Headache Rep 18:437.
183. Kim H, Shim JW, et al (2023). Korean Medicine Clinical Practice Guideline Update for Temporomandibular Disorders: An Evidence-Based Approach Healthcare (Switzerland) 11.
184. Kim KW, Ha IH, et al (2018). A clinical practice guideline for temporomandibular disorders in traditional Korean medicine: An evidence-based approach European Journal of Integrative Medicine 23:123-33.
185. Koh H, Robinson PG (2016). WITHDRAWN: Occlusal adjustment for treating and preventing temporomandibular joint disorders Cochrane Database Syst Rev Cd003812.
186. Kotiranta U, Suvinen T, et al (2014). Tailored treatments in temporomandibular disorders: where are we now? A systematic qualitative literature review J Oral Facial Pain Headache 28:28-37.
187. Kulkarni S, Thambar S, et al (2020). Evaluating the effectiveness of nonsteroidal anti-inflammatory drug(s) for relief of pain associated with temporomandibular joint disorders: A systematic review Clin Exp Dent Res 6:134-46.
188. La Touche R, Angulo-Díaz-Parreño S, et al (2010a). Effectiveness of acupuncture in the treatment of temporomandibular disorders of muscular origin: a systematic review of the last decade J Altern Complement Med 16:107-12.
189. La Touche R, Boo-Mallo T, et al (2022). Manual therapy and exercise in temporomandibular joint disc displacement without reduction. A systematic review Cranio 40:440-50.
190. La Touche R, Goddard G, et al (2010b). Acupuncture in the treatment of pain in temporomandibular disorders: a systematic review and meta-analysis of randomized controlled trials Clin J Pain 26:541-50.
191. La Touche R, Martínez García S, et al (2020). Effect of Manual Therapy and Therapeutic Exercise Applied to the Cervical Region on Pain and Pressure Pain Sensitivity in Patients with Temporomandibular Disorders: A Systematic Review and Meta-analysis Pain Med 21:2373-84.
192. Lam AC, Liddle LJ, et al (2023). The Effect of Upper Cervical Mobilization/Manipulation on Temporomandibular Joint Pain, Maximal Mouth Opening, and Pressure Pain Thresholds: A Systematic Review and Meta-Analysis Arch Rehabil Res Clin Transl 5:100242.
193. Langaliya A, Alam MK, et al (2023). Occurrence of Temporomandibular Disorders among patients undergoing treatment for Obstructive Sleep Apnoea Syndrome (OSAS) using Mandibular Advancement Device (MAD): A Systematic Review conducted according to PRISMA guidelines and the Cochrane handbook for systematic reviews of interventions J Oral Rehabil 50:1554-63.
194. Law D, McDonough S, et al (2015). Laser Acupuncture for Treating Musculoskeletal Pain: A Systematic Review with Meta-analysis JAMS Journal of Acupuncture and Meridian Studies 8:2-16.
195. Lee NW, Lee SH, et al (2023). Effectiveness of Chuna (or Tuina) Manual Therapy for Temporomandibular Disorder: A Systematic Review Altern Ther Health Med 29:258-68.
196. Leite FMG, Atallah Á, et al (2009). Cyclobenzaprine for the treatment of myofascial pain in adults Cochrane Database of Systematic Reviews.
197. Leite RA, Rodrigues JF, et al (2013). Relationship between temporomandibular disorders and orthodontic treatment: a literature review Dental press j. orthod. (Impr.) 18:150-7.
198. Leung YY, Wu FHW, et al (2020). Ultrasonography-guided arthrocentesis versus conventional arthrocentesis in treating internal derangement of temporomandibular joint: a systematic review Clin Oral Investig 24:3771-80.
199. Li C, Zhang Y, et al (2012). Inferior or double joint spaces injection versus superior joint space injection for temporomandibular disorders: a systematic review and meta-analysis J Oral Maxillofac Surg 70:37-44.
200. Li CJ, Shi ZD (2011). Something different from an updating Cochrane systematic review to a published systematicreview Cranio : the journal of craniomandibular practice 29:11; author reply 2-3.
201. Li DTS, Wong NSM, et al (2021). Timing of arthrocentesis in the management of temporomandibular disorders: an integrative review and meta-analysis Int J Oral Maxillofac Surg 50:1078-88.
202. Li J, Chen H (2023). Intra-articular injection of platelet-rich plasma vs hyaluronic acid as an adjunct to TMJ arthrocentesis: A systematic review and meta-analysis J Stomatol Oral Maxillofac Surg 101676.
203. Li J, Zhang Z, et al (2022). Diverse therapies for disc displacement of temporomandibular joint: a systematic review and network meta-analysis Br J Oral Maxillofac Surg 60:1012-22.
204. Liapaki A, Thamm JR, et al (2021). Is there a difference in treatment effect of different intra-articular drugs for temporomandibular joint osteoarthritis? A systematic review of randomized controlled trials Int J Oral Maxillofac Surg 50:1233-43.
205. Liberato FM, da Silva TV, et al (2023). Manual Therapy Applied to the Cervial Joint Reduces Pain and Improves Jaw Function in Individuals with Temporomandibular Disorders: A Systematic Review on Manual Therapy for Orofacial Disorders J Oral Facial Pain Headache 37:101-11.
206. Lima ECBd, Gonçalves EC, et al (2004). Treino de postura em pacientes portadores de disfunçõestemporomandibulares Reabilitar 6:55-9.
207. Lima RBWe, Cardoso AMR, et al (2013). Ortodontia como tratamento da disfunção temporomandibular: determinação do nível de evidência científica da literatura Rev. bras. ciênc. saúde 17:97-104.
208. List T, Axelsson S (2010). Management of TMD: evidence from systematic reviews and meta-analyses J Oral Rehabil 37:430-51.
209. List T, Axelsson S, et al (2003). Pharmacologic interventions in the treatment of temporomandibular disorders, atypical facial pain, and burning mouth syndrome. A qualitative systematic review J Orofac Pain 17:301-10.
210. Liu GF, Gao Z, et al (2021a). Effects of Warm Needle Acupuncture on Temporomandibular Joint Disorders: A Systematic Review and Meta-Analysis of Randomized Controlled Trials Evid Based Complement Alternat Med 2021:6868625.
211. Liu S, Hu Y, et al (2021b). Do intra-articular injections of analgesics improve outcomes after temporomandibular joint arthrocentesis?: A systematic review and meta-analysis J Oral Rehabil 48:95-105.
212. Liu Y, Wu J, et al (2018). Is There a Difference in Intra-Articular Injections of Corticosteroids, Hyaluronate, or Placebo for Temporomandibular Osteoarthritis? J Oral Maxillofac Surg 76:504-14.
213. Liu Y, Cao Y, et al (2012). Effectiveness of manual therapy for temporomandibular disorders: A systematic review of randomized controlled trials. Journal of Oral Rehabilitation 39:786-97.
214. Luo LY, Lee J, et al (2023). Psychological Outcomes on Anxiety and Depression after Interventions for Temporomandibular Disorders: A Systematic Review and Meta-Analysis Diagnostics (Basel) 13.
215. Luther F, Layton S, et al (2016). WITHDRAWN: Orthodontics for treating temporomandibular joint (TMJ) disorders Cochrane Database Syst Rev Cd006541.
216. Machado D, Martimbianco ALC, et al (2020). Botulinum Toxin Type A for Painful Temporomandibular Disorders: Systematic Review and Meta-Analysis J Pain 21:281-93.
217. Machado E, Bonotto D, et al (2013). Intra-articular injections with corticosteroids and sodium hyaluronate for treating temporomandibular joint disorders: a systematic review Dental Press J Orthod 18:128-33.
218. Machado E, dos Santos LZ, et al (2012a). Botulinum toxin for treating muscular temporomandibular disorders: A systematic review Dental Press Journal of Orthodontics 17:167-71.
219. Machado E, Machado P, et al (2012b). Use of chondroitin sulphate and glucosamine sulphate in degenerative changes in TMJ: A systematic review Dental Press Journal of Orthodontics 17:19.e1-.e5.
220. Machado E, Machado P, et al (2012c). Orthodontics as a therapeutic option for temporomandibular disorders: A systematic review Dental Press Journal of Orthodontics 17:98-102.
221. Machado E, Machado P, et al (2018). A systematic review of different substance injection and dry needling for treatment of temporomandibular myofascial pain Int J Oral Maxillofac Surg 47:1420-32.
222. Machado NAdG, Lima FF, et al (2014). Current panorama of temporomandibular disorders&#039; field in Brazil J. appl. oral sci 22:146-51.
223. Maia ML, Bonjardim LR, et al (2012). Effect of low-level laser therapy on pain levels in patients with temporomandibular disorders: a systematic review J Appl Oral Sci 20:594-602.
224. Maluf SA, Moreno BGD, et al (2008). Exercícios terapêuticos nas desordens temporomandibulares: uma revisão de literatura Fisioter. pesqui 15:408-15.
225. Manfredini D, Piccotti F, et al (2010). Hyaluronic acid in the treatment of TMJ disorders: a systematic review of the literature Cranio 28:166-76.
226. Manfredini D, Poggio CE (2017). Prosthodontic planning in patients with temporomandibular disorders and/or bruxism: A systematic review J Prosthet Dent 117:606-13.
227. Manrriquez SL, Robles K, et al (2021). Reduction of headache intensity and frequency with maxillary stabilization splint therapy in patients with temporomandibular disorders-headache comorbidity: a systematic review and meta-analysis J Dent Anesth Pain Med 21:183-205.
228. Marlière DAA, Vicentin Calori MJA, et al (2023). Clinical outcomes of the discopexy using suture anchors for repositioning disc displacement in temporomandibular joints: Systematic review and meta-analysis J Craniomaxillofac Surg 51:475-84.
229. Martin WJ, Forouzanfar T (2011). The efficacy of anticonvulsants on orofacial pain: a systematic review Oral Surg Oral Med Oral Pathol Oral Radiol Endod 111:627-33.
230. Martin WJ, Perez RS, et al (2012). Efficacy of antidepressants on orofacial pain: a systematic review Int J Oral Maxillofac Surg 41:1532-9.
231. Martins WR, Blasczyk JC, et al (2016). Efficacy of musculoskeletal manual approach in the treatment of temporomandibular joint disorder: A systematic review with meta-analysis Man Ther 21:10-7.
232. Máximo C, Coêlho JF, et al (2022). Effects of low-level laser photobiomodulation on the masticatory function and mandibular movements in adults with temporomandibular disorder: a systematic review with meta-analysis CoDAS 34:e20210138.
233. McNeely ML, Armijo Olivo S, et al (2006). A systematic review of the effectiveness of physical therapy interventions for temporomandibular disorders Phys Ther 86:710-25.
234. McQuay H, Carroll D, et al (1995). Anticonvulsant drugs for management of pain: a systematic review Bmj 311:1047-52.
235. Medlicott MS, Harris SR (2006). A systematic review of the effectiveness of exercise, manual therapy, electrotherapy, relaxation training, and biofeedback in the management of temporomandibular disorder Phys Ther 86:955-73.
236. Mehraban SH, Jamali S, et al (2020). Evaluating the effectiveness of orthognathic surgery on the pre-existing temporomandibular disorders in patients with malocclusion: A systematic review and meta-analysis Pesquisa Brasileira em Odontopediatria e Clinica Integrada 20:1-8.
237. Melis M, Di Giosia M, et al (2012). Low level laser therapy for the treatment of temporomandibular disorders: a systematic review of the literature Cranio 30:304-12.
238. Melis M, Di Giosia M, et al (2022). Oral myofunctional therapy for the treatment of temporomandibular disorders: A systematic review Cranio 40:41-7.
239. Melo AR, Pereira Júnior ED, et al (2017). Recurrent dislocation: scientific evidence and management following a systematic review Int J Oral Maxillofac Surg 46:851-6.6
240. Melo G, Casett E, et al (2018). Effects of glucosamine supplements on painful temporomandibular joint osteoarthritis: A systematic review J Oral Rehabil 45:414-22.
241. Melo MMS, Pataro SMS (2018). Eficácia da reeducação postural global na dor de indivíduos com disfunção temporomandibular: uma revisão sistemática Rev. Pesqui. Fisioter 8:437-47.
242. Mélou C, Sixou JL, et al (2023). Temporomandibular disorders in children and adolescents: A review Archives de Pediatrie 30:335-42.
243. Mena M, Dalbah L, et al (2020). Efficacy of topical interventions for temporomandibular disorders compared to placebo or control therapy: a systematic review with meta-analysis J Dent Anesth Pain Med 20:337-56.
244. Menéndez-Torre Á, Pintado-Zugasti AM, et al (2023). Effectiveness of deep dry needling versus manual therapy in the treatment of myofascial temporomandibular disorders: a systematic review and network meta-analysis Chiropr Man Therap 31:46.
245. Merz AE, Campus G, et al (2022). Hypnosis on acute dental and maxillofacial pain relief: A systematic review and meta-analysis J Dent 123:104184.
246. Milutka YA, Dyachkova YY, et al (2023). Results of the osteopathic treatment of the temporomandibular joint dysfunction (meta-analysis) Rossijskij Osteopaticeskij Zurnal 2023:95-108.
247. Minervini G, Franco R, et al (2023a). Conservative treatment of temporomandibular joint condylar fractures: A systematic review conducted according to PRISMA guidelines and the Cochrane Handbook for Systematic Reviews of Interventions J Oral Rehabil 50:886-93.
248. Minervini G, Marrapodi MM, et al (2023b). Temporomandibular disorders and orofacial neuropathic pain in children and adolescents: a systematic review J Clin Pediatr Dent 47:26-38.
249. Mittal N, Goyal M, et al (2019). Outcomes of surgical management of TMJ ankylosis: A systematic review and meta-analysis. J Craniomaxillofac Surg. 47(7):1120-1133.
250. Moldez MA, Camones VR, et al (2018). Effectiveness of Intra-Articular Injections of Sodium Hyaluronate or Corticosteroids for Intracapsular Temporomandibular Disorders: A Systematic Review and Meta-Analysis J Oral Facial Pain Headache 32:53–66.
251. Monje-Gil F, Nitzan D, et al (2012). Temporomandibular joint arthrocentesis. Review of the literature Medicina Oral, Patologia Oral y Cirugia Bucal 17:e575-e81.
252. Monteiro J, de Arruda JAA, et al (2020). Is Single-Puncture TMJ Arthrocentesis Superior to the Double-Puncture Technique for the Improvement of Outcomes in Patients With TMDs? J Oral Maxillofac Surg 78:1319.e1-.e15.
253. Monteiro J, Guastaldi FPS, et al (2021). Induction, Treatment, and Prevention of Temporomandibular Joint Ankylosis-A Systematic Review of Comparative Animal Studies J Oral Maxillofac Surg 79:109-32.e6.
254. Montinaro F, Nucci L, et al (2022). Oral nonsteroidal anti-inflammatory drugs as treatment of joint and muscle pain in temporomandibular disorders: A systematic review Cranio 1-10.
255. Moon TW, Choi TY, et al (2013). Chuna therapy for musculoskeletal pain: a systematic review of randomized clinical trials in Korean literature Chin J Integr Med 19:228-32.
256. Moraes AdR, Sanches ML, et al (2013). Therapeutic exercises for the control of temporomandibular disorders Dental press j. orthod. (Impr.) 18:134-9.
257. Morales H (1992). Disfunción de la articulación temporo mandibular Odontol. día 9:12-8.
258. Morales Trejo B (2003). Evaluación y conceptos de artrocentesis Rev. ADM 60:25-8.
259. Moussa MS, Bachour D, et al (2023). Adverse effect of botulinum toxin-A injections on mandibular bone: A systematic review and meta-analysis J Oral Rehabil.
260. Müggenborg F, de Castro Carletti EM, et al (2023). Effectiveness of Manual Trigger Point Therapy in Patients with Myofascial Trigger Points in the Orofacial Region-A Systematic Review Life (Basel) 13.
261. Mujakperuo HR, Watson M, et al (2010). Pharmacological interventions for pain in patients with temporomandibular disorders Cochrane Database Syst Rev Cd004715.
262. Munguia FM, Jang J, et al (2018). Efficacy of Low-Level Laser Therapy in the Treatment of Temporomandibular Myofascial Pain: A Systematic Review and Meta-Analysis J Oral Facial Pain Headache 32:287–97.
263. Naeije M, Te Veldhuis AH, et al (2013). Disc displacement within the human temporomandibular joint: a systematic review of a 'noisy annoyance' J Oral Rehabil 40:139-58.
264. Nagori SA, Bansal A, et al (2021). Comparison of outcomes with the single-puncture and double-puncture techniques of arthrocentesis of the temporomandibular joint: An updated systematic review and meta-analysis J Oral Rehabil 48:1056-65.
265. Nagori SA, Roy Chowdhury SK, et al (2018). Single puncture versus standard double needle arthrocentesis for the management of temporomandibular joint disorders: A systematic review J Oral Rehabil 45:810-8.
266. Naik PN, Kiran RA, et al (2014). Acupuncture: An alternative therapy in dentistry and its possible applications Medical Acupuncture 26:308-14.
267. Nandhini J, Ramasamy S, et al (2018). Is nonsurgical management effective in temporomandibular joint disorders? – A systematic review and meta-analysis Dent Res J (Isfahan) 15:231-41.
268. Netto BdP, Maior BSS, et al (2007). Laserterapia de baixa intensidade no tratamento de desordens temporomandibulares Rev. Fac. Odontol. Porto Alegre 48:88-91.
269. Niezen ET, van Minnen B, et al (2023). Temporomandibular joint prosthesis as treatment option for mandibular condyle fractures: a systematic review and meta-analysis Int J Oral Maxillofac Surg 52:88-97.
270. Nimonkar S, Godbole S, et al (2022). Effect of Rehabilitation of Completely Edentulous Patients With Complete Dentures on Temporomandibular Disorders: A Systematic Review Cureus 14:e28012.
271. Nowak Z, Chęciński M, et al (2021). Intramuscular Injections and Dry Needling within Masticatory Muscles in Management of Myofascial Pain. Systematic Review of Clinical Trials Int J Environ Res Public Health 18.
272. Olate S, Ravelo V, et al (2023). An Overview of Clinical Conditions and a Systematic Review of Personalized TMJ Replacement Journal of Personalized Medicine 13.
273. Ortiz G, Quito E (2022). Efectividad de la terapia manual en trastornos temporomandibulares. Revisión de literatura Odontol. sanmarquina (Impr.) 25:e22075-e.
274. Owen M, Gray B, et al (2022). Impact of botulinum toxin injection into the masticatory muscles on mandibular bone: A systematic review J Oral Rehabil 49:644-53.
275. Paço M, Peleteiro B, et al (2016). The Effectiveness of Physiotherapy in the Management of Temporomandibular Disorders: A Systematic Review and Meta-analysis J Oral Facial Pain Headache 30:210-20.
276. Palmer J, Penlington C, et al (2023). Supported self-management in temporomandibular disorders: A systematic review of behavioural components Oral Surgery 16:228-36.
277. Pan G, Li C, et al (2012). Application of dental chairside evidence-based medicine for the treatment temporomandibular osteoarthritis Hua xi kou qiang yi xue za zhi = Huaxi kouqiang yixue zazhi = West China journal of stomatology 30:624-7, 31.
278. Park EY, Cho JH, et al (2023). Is acupuncture an effective treatment for temporomandibular disorder?: A systematic review and meta-analysis of randomized controlled trials Medicine (Baltimore) 102:e34950.
279. Patel J, Cardoso JA, et al (2019). A systematic review of botulinum toxin in the management of patients with temporomandibular disorders and bruxism Br Dent J 226:667-72.
280. Peixoto KO, Abrantes PS, et al (2023). Temporomandibular disorders and the use of traditional and laser acupuncture: a systematic review Cranio 41:501-7.
281. Penlington C, Senthil K, et al (2022). Self-management for chronic orofacial pain: A systematic review of randomized controlled trials. Journal of Oral Rehabilitation 49:554-66.
282. Penlington C, Otemade AA,et al (2019). Psychological therapies for temporomandibular disorders (TMD). Cochrane Database of Systematic Reviews 2019, Issue 12.
283. Pereira IN, Hassan H (2022). Botulinum toxin A in dentistry and orofacial surgery: an evidence-based review - part 1: therapeutic applications Evidence-Based Dentistry.
284. Petrucci A, Sgolastra F, et al (2011). Effectiveness of low-level laser therapy in temporomandibular disorders: a systematic review and meta-analysis J Orofac Pain 25:298-307.
285. Pimentel AM, Magno MB, et al (2018). Does physical therapy reduce pain and improve range of motion in patients with temporomandibular disorders? A systematic review with meta-analysis. Journal of Oral Rehabilitation 45:753-63.
286. Pimentel de França AM, Gonçalves Borges A, et al (2021). Photobiomodulation in temporomandibular dysfunction: A systematic review Muscles, Ligaments and Tendons Journal 11:463-74.
287. Pliavga V, Šakalys D, et al (2022). The Efficiency of Different Arthroscopic Discopexy Techniques Based on Clinical and Radiographic Findings: A Systematic Review J Craniofac Surg 33:e714-e9.
288. Porporatti AL, Costa YM, et al (2019). Placebo and nocebo response magnitude on temporomandibular disorder-related pain: A systematic review and meta-analysis J Oral Rehabil 46:862-82.
289. Prado-Posada S, Da Cuña-Carrera I, et al (2020). Physiotherapy in temporomandibular disorders: A systematic review Medicina Naturista 14:79-85.
290. Prechel U, Ottl P, et al (2018). The treatment of temporomandibular joint dislocation - A systematic review Deutsches Arzteblatt International 115:59-64.
291. Priyank H, Shankar Prasad R, et al (2023). Management protocols of chronic Orofacial Pain: A Systematic Review Saudi Dental Journal 35:395-402.
292. Quinelato V, Balduino A, et al (2011). Arnica montana e desordens musculares mastigatórias Rev. bras. Odontol 68:225-8.
293. Raman S, Ikutame D, et al (2023). Targeted Therapy for Orofacial Pain: A Novel Perspective for Precision Medicine Journal of Personalized Medicine 13.
294. Ramos-Herrada RM, Arriola-Guillén LE, et al (2022). Effects of botulinum toxin in patients with myofascial pain related to temporomandibular joint disorders: A systematic review Dent Med Probl 59:271-80.
295. Randhawa K, Bohay R, et al (2016). The effectiveness of noninvasive interventions for temporomandibular disorders a systematic review by the Ontario protocol for traffic injury management (OPTIMa) collaboration Clinical Journal of Pain 32:260-78.
296. Reginster JY, Bruyere O, et al (2007). Current role of glucosamine in the treatment of osteoarthritis Rheumatology 46:731-5.
297. Ren H, Liu J, et al (2022). Comparative effectiveness of low-level laser therapy with different wavelengths and transcutaneous electric nerve stimulation in the treatment of pain caused by temporomandibular disorders: A systematic review and network meta-analysis J Oral Rehabil 49:138-49.
298. Rigon M, Pereira LM, et al (2015). WITHDRAWN: Arthroscopy for temporomandibular disorders Cochrane Database Syst Rev Cd006385.
299. Riley P, Glenny AM, et al (2020). Oral splints for temporomandibular disorder or bruxism: a systematic review Br Dent J 228:191-7.
300. Rinchuse DJ, McMinn JT (2006). Summary of evidence-based systematic reviews of temporomandibular disorders American Journal of Orthodontics and Dentofacial Orthopedics 130:715-20.
301. Rizzatti-Barbosa CM, Albergaria-Barbosa JRd (2018). Atualidades sobre o tratamento das disfunções temporomandibulares ImplantNewsPerio 3:138-46.
302. Rodhen RM, de Holanda TA, et al (2022). Invasive surgical procedures for the management of internal derangement of the temporomandibular joint: a systematic review and meta-analysis regarding the effects on pain and jaw mobility Clin Oral Investig 26:3429-46.
303. Roldán-Barraza C, Janko S, et al (2014). A systematic review and meta-analysis of usual care and psychosocial interventions in the treatment of myofascial temporomandibular disorder pain. Journal of Oral & Facial Pain and Headache 28:205-22.
304. Rosted P (1998). The use of acupuncture in dentistry: A systematic review Acupuncture in Medicine 16:43-8.
305. Ruiz-Romero V, Toledano-Serrabona J, et al (2022). Efficacy of the use of chondroitin sulphate and glucosamine for the treatment of temporomandibular joint dysfunction: A systematic review and meta-analysis Cranio 1-10.
306. Sàbado-Bundó H, Sánchez-Garcés M, et al (2021). Intraarticular injections of hyaluronic acid in arthrocentesis and arthroscopy as a treatment of temporomandibular joint disorders: A systematic review Cranio 1-10.
307. Sakalys D, Dvylys D, et al (2020). Comparison of Different Intraarticular Injection Substances Followed by Temporomandibular Joint Arthroscopy J Craniofac Surg 31:637-41.
308. Salami AA, Kanmodi KK, et al (2023). The use of Qigong and Tai Chi in the management of temporomandibular joint dysfunction: A systematic review Health Sci Rep 6:e1639.
309. Sales JM, Lavôr TFAd, et al (2020). Toxina botulínica como opção no tratamento da disfunção temporomandibular Rev. Salusvita (Online) 39:229-54.
310. Sánchez Torres J, Toranzo Fernández JM (1994). Patología quirúrgica de la articulación temporomandibular Rev. ADM 51:217-25.7
311. Sandoval GP, Yepes A, et al (1997). Articulación temporomandibular: síndromes dolorosos miofaciales Acta otorrinolaringol. cir. cabeza cuello 25:35-40.
312. Santander H, Santander MC, et al (2011). Después de cien años de uso: ¿las férulas oclusales tienen algún efecto terapéutico? Rev. clín. periodoncia implantol. rehabil. oral (Impr.) 4:29-35.
313. Santonocito S, Donzella M, et al (2023). Orofacial Pain Management: An Overview of the Potential Benefits of Palmitoylethanolamide and Other Natural Agents Pharmaceutics 15.
314. Santos TS, Pagotto LEC, et al (2021). Effectiveness of disk repositioning and suturing comparing open-joint versus arthroscopic techniques: a systematic review and meta-analysis Oral Surg Oral Med Oral Pathol Oral Radiol 132:506-13.
315. Saraiva L, Adriana Campos Tortelli S, et al (2019). Tratamento de disfunção temporomandibular com ozonioterapia: revisão sistemática RFO UPF 24;:316-21.
316. Saranya SK, Janakiram C, et al (2019). Efficacy of occlusal splints in managing temporomandibular disorders Indian Journal of Public Health Research and Development 10:464-9.
317. Sassi FC, Silva APd, et al (2018). Tratamento para disfunções temporomandibulares: uma revisão sistemática Audiol.,Commun. res 23:e1871-e.
318. Schindler HJ, Türp JC, et al (2007). Therapy of masticatory muscle pain. Recommendations for clinical management Schmerz 21:102-15.
319. Sembronio S, Tel A, et al (2021). The use of cutting/positioning devices for custom-fitted temporomandibular joint alloplastic reconstruction: current knowledge and development of a new system International Journal of Oral and Maxillofacial Surgery 50:530-7.
320. Senye M, Mir CF, et al (2012). Topical nonsteroidal anti-inflammatory medications for treatment of temporomandibular joint degenerative pain: a systematic review J Orofac Pain 26:26-32.
321. Serrano-Muñoz D, Beltran-Alacreu H, et al (2023). Effectiveness of Different Electrical Stimulation Modalities for Pain and Masticatory Function in Temporomandibular Disorders: A Systematic Review and Meta-Analysis J Pain 24:946-56.
322. Shimada A, Ishigaki S, et al (2023). Effects of self-care education on patients with temporomandibular disorders: A systematic review and meta-analysis. Journal of Prosthodontic Research 67:475-84.
323. Serrera-Figallo MA, Ruiz-De-León-Hernández G, et al (2020). Use of botulinum toxin in orofacial clinical practice Toxins 12.
324. Shaffer SM, Brismée JM, et al (2014). Temporomandibular disorders. Part 2: Conservative management Journal of Manual and Manipulative Therapy 22:13-23.
325. Sharma P, Singh G, et al (2022). Effects of Manual Therapy in Somatic Tinnitus Patients Associated with Cervicogenic and Temporomandibular Dysfunction Domain: Systematic Review and Meta Analysis of Randomised Controlled Trials Indian J Otolaryngol Head Neck Surg 74:247-53.
326. Shi Z, Guo C, et al (2013). WITHDRAWN: Hyaluronate for temporomandibular joint disorders Cochrane Database Syst Rev Cd002970.
327. Shukla D, Muthusekhar MR (2016). Efficacy of low-level laser therapy in temporomandibular disorders: A systematic review Natl J Maxillofac Surg 7:62-6.
328. Siewert-Gutowska M, Pokrowiecki R, et al (2023). State of the Art in Temporomandibular Joint Arthrocentesis-A Systematic Review J Clin Med 12.
329. Silva YSd, Grillo R (2011). Injeções intra-articulares de corticosteróides versus hialuronato de sódio para tratamento de desarranjos internos da articulação temporomandibular Odonto (Säo Bernardo do Campo) 19:105-14.
330. Singh AK, Jose A, et al (2022). Transport distraction osteogenesis compared with autogenous grafts for ramus-condyle unit reconstruction in temporomandibular joint ankylosis: a systematic review and meta-analysis Br J Oral Maxillofac Surg 60:731-9.
331. Singh BP, Jayaraman S, et al (2017). Occlusal interventions for managing temporomandibular disorders. Cochrane Database of Systematic Reviews 2017, Issue 11.
332. Singh JA (2013). Use of botulinum toxin in musculoskeletal pain F1000Research 2.
333. Sit RW, Reeves KD, et al (2021). Efficacy of hypertonic dextrose injection (prolotherapy) in temporomandibular joint dysfunction: a systematic review and meta-analysis Sci Rep 11:14638.
334. Sobral AP, Sobral SS, et al (2021). Photobiomodulation and myofascial temporomandibular disorder: Systematic review and meta-analysis followed by cost-effectiveness analysis J Clin Exp Dent 13:e724-e32.
335. Sommer C (2002). [Pharmacotherapy of orofacial pain] Schmerz 16:381-8.
336. Song YL, Yap AU (2018). Outcomes of therapeutic TMD interventions on oral health related quality of life: A qualitative systematic review Quintessence Int 49:487-96.
337. Soviero VM, Gama FVda, et al (1997). Disfunçäo da articulaçäo têmporo-mandibular em crianças: revisäo de literatura J. bras. ortodontia ortop. maxilar 2:49-52.
338. Sposito MMdM, Teixeira SAF (2014). Toxina Botulínica Tipo A no tratamento da dor miofascial relacionada aos músculos da mastigação Acta fisiátrica 21.
339. Story WV, Chichirez CM, et al (2016). Self-care management in patients with temporomandibular disorders: A systematic review. Journal of Medicine and Life 9:366-70.
340. Sterniczuk B, Rossouw PE, et al (2022). Effectiveness of Curcumin in Reducing Self-Rated Pain-Levels in the Orofacial Region: A Systematic Review of Randomized-Controlled Trials Int J Environ Res Public Health 19.
341. Stoustrup P, Kristensen KD, et al (2013). Intra-articular steroid injection for temporomandibular joint arthritis in juvenile idiopathic arthritis: A systematic review on efficacy and safety. Semin Arthritis Rheum. 43(1):63-70.
342. Sung SH, Kim D, et al (2021). Electroacupuncture for Temporomandibular Disorders: A Systematic Review of Randomized Controlled Trials Healthcare (Basel) 9.
343. Te Veldhuis EC, Te Veldhuis AH, et al (2017). The effect of orthognathic surgery on the temporomandibular joint and oral function: a systematic review Int J Oral Maxillofac Surg 46:554-63.
344. Tengrungsun T, Mitrirattanakul S, et al (2012). Is low level laser effective for the treatment of orofacial pain?: A systematic review Cranio 30:280-5.
345. Tesch RS, Macedo L, et al (2021). Effectiveness of dry needling on the local pressure pain threshold in patients with masticatory myofascial pain. Systematic review and preliminary clinical trial Cranio 39:171-9.
346. Thambar S, Kulkarni S, et al (2020). Botulinum toxin in the management of temporomandibular disorders: a systematic review Br J Oral Maxillofac Surg 58:508-19.
347. Thorpe A, Haddad Y, et al (2023). A systematic review and meta-analysis of randomized controlled trials comparing arthrocentesis with conservative management for painful temporomandibular joint disorder Int J Oral Maxillofac Surg 52:889-96.
348. Tocaciu S, McCullough MJ, et al (2019). Surgical management of recurrent TMJ dislocation-a systematic review Oral Maxillofac Surg 23:35-45.
349. Torres D, Zaror C, et al (2020). Intra-articular corticosteroids for treatment of temporomandibular joint internal disorders: protocol for systematic review and network meta-analysis. BMJ Open. 10(9):e034327.
350. Torres-Rosas R, Marcela Castro-Gutiérrez ME, et al (2023). Ozone for the treatment of temporomandibular joint disorders: a systematic review and meta-analysis Med Gas Res 13:149-54.
351. Tournavitis A, Sandris E, et al (2023). Effectiveness of conservative therapeutic modalities for temporomandibular disorders-related pain: a systematic review Acta Odontol Scand 81:286-97.
352. Tsui HC, Lam CM, et al (2022). Lavage Volume of Arthrocentesis in the Management of Temporomandibular Disorders: A Systematic Review and Meta-Analysis Diagnostics (Basel) 12.
353. Tunér J, Hosseinpour S, et al (2019). Photobiomodulation in Temporomandibular Disorders Photobiomodul Photomed Laser Surg 37:826-36.
354. Türp JC, Jokstad A, et al (2007a). Is there a superiority of multimodal as opposed to simple therapy in patients with temporomandibular disorders? A qualitative systematic review of the literature Clin Oral Implants Res 18 Suppl 3:138-50.
355. Türp JC, Motschall E, et al (2007b). In patients with temporomandibular disorders, do particular interventions influence oral health-related quality of life? A qualitative systematic review of the literature Clin Oral Implants Res 18 Suppl 3:127-37.
356. Undabarrena IU, González YG, et al (2020). Effectiveness of stretching in musculoskeletal diseases: A systematic review Medicina Naturista 14:107-16.
357. Valladares-Neto J, Cevidanes LH, et al (2014). TMJ response to mandibular advancement surgery: an overview of risk factors J. appl. oral sci 22:2-14.
358. Vallejo-Rosero K, Lanas-Teran G, et al (2019). Is low-level laser therapy (LLLT) effective in relieving the symptoms of temporomandibular disorders (TMDs)? A systematic review Rev. Fac. Odontol. Univ. Antioq 31:136-46.
359. van der Meer HA, Calixtre LB, et al (2020). Effects of physical therapy for temporomandibular disorders on headache pain intensity: A systematic review Musculoskelet Sci Pract 50:102277.
360. van Selms MK, Naeije M, et al (2009). [Myogenous temporomandibular pain: treat with care!] Ned Tijdschr Tandheelkd 116:260-5.
361. Varedi P, Bohluli B (2015). Autologous blood injection for treatment of chronic recurrent TMJ dislocation: is it successful? Is it safe enough? A systematic review Oral Maxillofac Surg 19:243-52.
362. Vasconcelos BCdE, Bessa-Nogueira RV, et al (2006). Artrocentese da articulação temporomandibular: avaliação de resultados e revisão da literatura Rev. bras. otorrinolaringol 72:634-8.
363. Vasconcelos BCdE, Porto GG, et al (2008). Anquilose da articulação têmporo-mandibular Rev. bras. otorrinolaringol 74:34-8.
364. Velasco C, Salazar de Plaza E (2003). Tratamiento farmacológico de los desórdenes temporomandibulares Acta odontol. venez 41:47-55.
365. Vergara Muñoz S (1996). Uso de la condilotomía modificada como alternativa de tratamiento para desplazamiento anterior del disco con reducción y sintomatología dolorosa de la ATM, pp. 268-.
366. Vidya VS, Sumathi Felicita A (2015). Efficacy of pharmacological agents in the treatment of temporomandibular joint disorder: A systematic review International Journal of Pharmacy and Pharmaceutical Sciences 7:54-8.
367. Vieira LS, Pestana PRM, et al (2023). The Efficacy of Manual Therapy Approaches on Pain, Maximum Mouth Opening and Disability in Temporomandibular Disorders: A Systematic Review of Randomised Controlled Trials Life (Basel) 13.
368. Vier C, Almeida MB, et al (2019). The effectiveness of dry needling for patients with orofacial pain associated with temporomandibular dysfunction: a systematic review and meta-analysis Braz J Phys Ther 23:3-11.
369. Vos LM, Huddleston Slater JJ, et al (2013). Lavage therapy versus nonsurgical therapy for the treatment of arthralgia of the temporomandibular joint: a systematic review of randomized controlled trials J Orofac Pain 27:171-9.
370. Votrubec C, Tran P, et al (2022). Cannabinoid therapeutics in orofacial pain management: a systematic review Aust Dent J 67:314-27.
371. Webb TR, Rajendran D (2016). Myofascial techniques: What are their effects on joint range of motion and pain? – A systematic review and meta-analysis of randomised controlled trials J Bodyw Mov Ther 20:682-99.
372. Wen S, Iturriaga V, et al (2023). Differences According to the Type of Exogenous Hyaluronic Acid and its Frequency of Infiltration in the Treatment of Temporomandibular Osteoarthritis Int. j. morphol 41:699-704.
373. Weyant RJ (2006). Questional benefit from occlusal adjustment for TMD disorders Journal of Evidence-Based Dental Practice 6:167-8.
374. Wiffen PJ (2011). Evidence-based pain management and palliative care in the october 2010 issue of the Cochrane library Journal of Pain and Palliative Care Pharmacotherapy 25:61-3.
375. Worasing K, Sungthong B, et al (2023). Effectiveness of Zingiber montanum Herbal Compress Remedy for Pain Management: An Updated Systematic Review and Meta-Analysis Scientia Pharmaceutica 91.
376. Wu X, Zhu J, et al (2021). Effectiveness of low-level gallium aluminium arsenide laser therapy for temporomandibular disorder with myofascial pain: A systemic review and meta-analysis Medicine (Baltimore) 100:e28015.
377. Xu GZ, Jia J, et al (2018). Low-Level Laser Therapy for Temporomandibular Disorders: A Systematic Review with Meta-Analysis Pain Res Manag 2018:4230583.
378. Xu J, Ren H, et al (2023). Comparative effectiveness of hyaluronic acid, platelet-rich plasma, and platelet-rich fibrin in treating temporomandibular disorders: a systematic review and network meta-analysis Head Face Med 19:39.
379. Zhang Y, Montoya L, et al (2015). Hypnosis/Relaxation therapy for temporomandibular disorders: a systematic review and meta-analysis of randomized controlled trials. Journal of Oral & Facial Pain and Headache 29:115-25.
380. Yaseen M, Abdulqader D, et al (2021). Temporomandibular Total Joint Replacement Implant Devices: A Systematic Review of Their Outcomes Journal of long-term effects of medical implants 31:91-8.
381. Zhang SH, He KX, et al (2020). Efficacy of occlusal splints in the treatment of temporomandibular disorders: a systematic review of randomized controlled trials Acta Odontol Scand 78:580-9.
382. Zhang XG, Yang F, et al (2008). Evidence of cochrane systematic reviews on the treatment of temporomandibular disorders Chinese Journal of Evidence-Based Medicine 8:1130-2.
383. Zhang Y, Qian Y, et al (2023). Efficacy of laser therapy for temporomandibular disorders: A systematic review and meta-analysis Complement Ther Med 74:102945.
384. Zheng Y, Liu CW, et al (2023). Neurostimulation for Chronic Pain: A Systematic Review of High-Quality Randomized Controlled Trials With Long-Term Follow-Up Neuromodulation 26:1276-94.
385. Zwiri A, Alrawashdeh MA, et al (2020). Effectiveness of the Laser Application in Temporomandibular Joint Disorder: A Systematic Review of 1172 Patients Pain Res Manag 2020:5971032.
